# Bioinspired Injectable Self-Healing Hydrogel Sealant with Fault-Tolerant and Repeated Thermo-Responsive Adhesion for Sutureless Post-Wound-Closure and Wound Healing

**DOI:** 10.1007/s40820-022-00928-z

**Published:** 2022-09-13

**Authors:** Yuqing Liang, Huiru Xu, Zhenlong Li, Aodi Zhangji, Baolin Guo

**Affiliations:** 1 State Key Laboratory for Mechanical Behavior of Materials, and Frontier Institute of Science and Technology, Xi’an Jiaotong University, Xi’an, 710049 People’s Republic of China; 2 Key Laboratory of Shaanxi Province for Craniofacial Precision Medicine Research, College of Stomatology, Xi’an Jiaotong University, Xi’an, 710049 People’s Republic of China

**Keywords:** Bioinspired injectable hydrogel, Tissue sealant, Temperature-dependent adhesion, Reversible adhesion, Wound healing

## Abstract

Hydrogels with multifunctionalities, including sufficient bonding strength, injectability and self-healing capacity, responsive-adhesive ability, fault-tolerant and repeated tissue adhesion, are urgently demanded for invasive wound closure and wound healing. Motivated by the adhesive mechanism of mussel and brown algae, bioinspired dynamic bonds cross-linked multifunctional hydrogel adhesive is designed based on sodium alginate (SA), gelatin (GT) and protocatechualdehyde, with ferric ions added, for sutureless post-wound-closure. The dynamic hydrogel cross-linked through Schiff base bond, catechol-Fe coordinate bond and the strong interaction between GT with temperature-dependent phase transition and SA, endows the resulting hydrogel with sufficient mechanical and adhesive strength for efficient wound closure, injectability and self-healing capacity, and repeated closure of reopened wounds. Moreover, the temperature-dependent adhesive properties endowed mispositioning hydrogel to be removed/repositioned, which is conducive for the fault-tolerant adhesion of the hydrogel adhesives during surgery. Besides, the hydrogels present good biocompatibility, near-infrared-assisted photothermal antibacterial activity, antioxidation and repeated thermo-responsive reversible adhesion and good hemostatic effect. The in vivo incision closure evaluation demonstrated their capability to promote the post-wound-closure and wound healing of the incisions, indicating that the developed reversible adhesive hydrogel dressing could serve as versatile tissue sealant.

**Supplementary Information:**

The online version contains supplementary material available at 10.1007/s40820-022-00928-z.

## Highlights

- Mussel and brown algae inspired dual-bionic bioadhesive cross-linked by dynamic bonds exhibits good self-healing capacity and sufficient adhesive strength. And the adhesive hydrogel shows temperature-dependent reversible adhesive behavior, and could achieve fault-tolerate adhesive application and repeated tissue adhesion.
- The coordination interaction between ferric ions and protocatechualdehyde equips the hydrogel with photothermal antibacterial effect.
- The injectable adhesive with antioxidation and good hemostatic effect shows promising application in tissue sealant and wound closure.

**Supplementary Information:**

The online version contains supplementary material available at 10.1007/s40820-022-00928-z.

## Graphical abstract

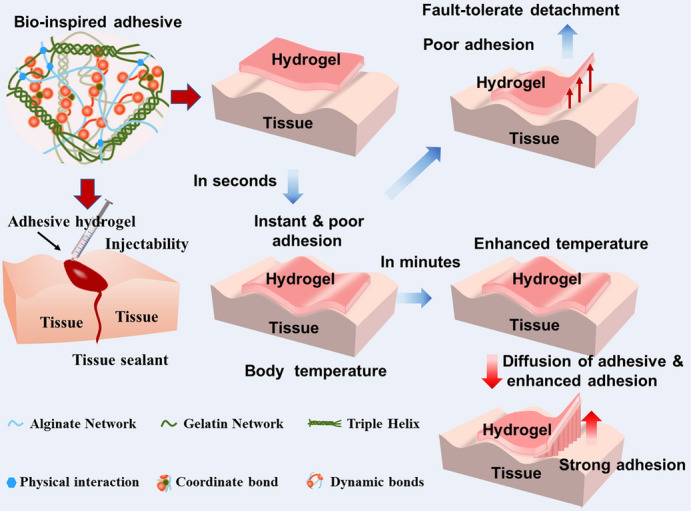

## Introduction

Wounds caused by accident or surgery are increasing each year, and severe open wounds require timely closure to ensure the healing of injuries [1]. However, traditional wound closure strategies, including suture and staple, are usually invasive, which might cause trauma, unsatisfied tissue integration and the leakage of the contents inside the sealed tissues due to the incomplete closure [1–4]. Moreover, the closed open wounds might take a long period, including inflammation, proliferation and remodeling phases, to recovery [5, 6]. Naturally, post-wound-closure care should be taken into consideration in the treatment of open wounds. Therefore, sutureless wound closure strategy, with less trauma and scar, less professional skill required and good patient compliance, has attracted lot of interests [7]. Rapid advances in materials science have shed lights on the design of biomedical functional materials [8–11], and various bioadhesives have been developed in recent years, which might be an ideal candidate for sutureless wound closure [12, 13]. Existing tissue sealants are usually divided into natural and synthetic adhesives. Natural sealants, take fibrin glue as an example, are widely accepted with good biocompatibility but weak adhesive strength and high cost. Moreover, viral infection is an issue that could not be ignored [1, 14]. Although synthetic sealants (e.g., cyanoacrylate glue) often show strong adhesion capability, they suffer from redundant preparation process, poor degradability and biocompatibility, bioinert, and the degraded components might cause inflammatory or toxicity response [15, 16]. Besides, almost all the synthetic sealants only could achieve one strong adhesion and might need the intervention of external stimuli (e.g., light, H_2_O), which is not conducive for secondary closure of reopened wounds due to the movement of body [4, 17–19]. And once covalent bonds are formed, the unavoidable mispositioning of adhesives on dynamic tissue surfaces could not be detached easily. Moreover, some external stimulus is difficult to impose when it comes to tissues with complex structures (e.g., spinal cord) [20]. Therefore, it is imperative to develop a versatile tissue sealant with sufficient bonding strength for efficient wound closure, injectability and self-healing capacity with facile operation and no external stimuli, fault-tolerant adhesion and secondary closure of reopened wounds, biocompatibility and bioactivity for post-wound-closure care.

**Fig. 1:**
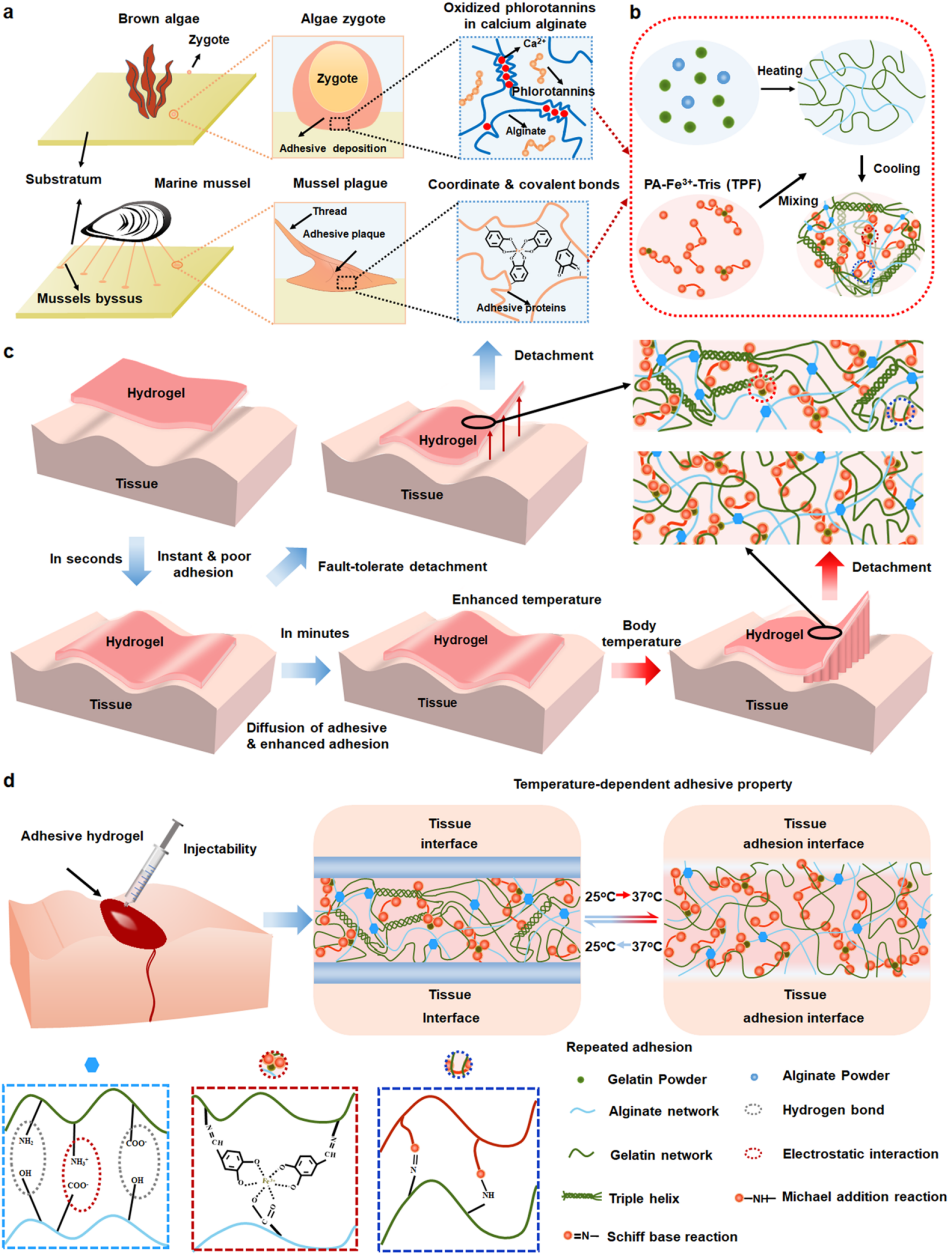
The schematic representations of the fault-tolerate detachment and temperature-responsive adhesive properties of the bioinspired adhesive hydrogel as tissue sealant. **a** Components of brown algae adhesives and marine mussel adhesives. **b** Schematic representations for the preparation of the bioinspired adhesive hydrogel. **c** The illustration for the fault-tolerate detachment of the hydrogel. The temperature-dependent adhesion is originated from the improved segment mobility of the GT due to the helix bundles in network transferred to random coils at body temperature, which increases the possibility of contact between the adhesives and the functional group. It might take several minutes for the temperature of the gel to warm up to body temperature, during which the mispositioning hydrogel could be removed and repositioned. **d** Schematic illustration for the temperature-dependent adhesive mechanism and repeated adhesion of the hydrogel as tissue sealant

Hydrogel, with controllable chemical and physical properties, high water content and biocompatibility, has attracted much attention in recent years [7, 21–29]. Adhesive hydrogels inspired by living systems, especially for various sessile marine organisms, have shown promising application in sutureless wound closure [1, 3, 30–37]. Many marine organisms could secrete mucilage containing mucins or polysaccharides, which could solidify in seawater [36, 38]. It is well documented that the adhesive mechanism of mussel is mainly due to the presence of dihydroxyphenylalanine (DOPA), lysine and hydroxyproline on byssus protein, and the high cross-linking capacity of mussel foot protein within its adhesion mechanism is involving the quinone moieties oxidized from catechol group in DOPA (Fig. 1a) [39–43]. And the catechol groups in mussel byssus protein could form pH-dependent coordinate bonds with ferric iron (Fe^3+^) to enhance underwater adhesion [1, 3]. Although mussel-inspired catechol chemistry has been extensively applied to develop strong adhesives [20, 30, 33, 44–47], and the mussel-inspired adhesives mainly focus on polymers grafted with catechol or polymerized from monomers modified with catechol group, and the recombinant mussel adhesive proteins included. Those sophisticated chemical modification is time-consuming and high-cost, and might need the intervention of a large amount of organic solvents, which limits their applications as biomedical materials. And the prepared strong sealants might adhere tightly to instruments during operation, which could cause great inconvenience to operators. Besides, it is reported that the sessile growth of brown algae is due to the adhesive mainly composed of calcium-alginate gel, phlorotannins and sulfated fucans, in which the numerous hydroxyl groups in phlorotannis promote the absorption and adhesion onto the substrate, and the calcium-alginate gel and the subsequent polymerization of phlorotannins ensure the cohesion of the adhesive (Fig. 1a) [35, 38]. And the brown algae adhesive strategy demonstrates that there is no need to require the presence of the phlorotannins on the polymer chain at the beginning [35]. However, there are few algal adhesion inspired adhesives due to the less clear adhesive mechanism and poor adhesion strength associated with phlorotannins-based adhesives (slightly stronger than pure calcium-alginate gel) [35]. Therefore, it is imperative to design and develop efficient sealant with responsive adhesive properties through facile strategy.

In this work, motivated by the reported adhesive mechanism of mussel and brown algae, we envisioned to develop a bioinspired dynamic bond cross-linked functional hydrogel with adhesive and mechanical strength sufficient for tissue sealant, injectability and responsive-adhesive ability with facile operation, fault-tolerant adhesion and repeated closure of reopened wounds, biocompatibility and bioactivity for post-wound-closure treatment (Fig. 1). Sodium alginate (SA), as the main component of the algae adhesive, could accommodate the following polyphenol aggregation with various chains and sizes, which plays an important role in the adhesion of brown algae [38]. Gelatin (GT), a protein with temperature-dependent phase transition, has good biocompatibility, degradability, and could promote tissue regeneration [2, 48–50]. The strong interaction between amino group and carboxyl group of GT and SA could endow the adhesives with cohesion. Inspired by the adhesive mechanism of mussel, catechol and aldehyde group contained protocatechualdehyde (PA) and Fe^3+^ were introduced into the sealant to further enhance the cohesion of the adhesives and accelerate the absorption and adhesion to the substrate (surface adhesion). Furthermore, quinone group oxidized from excess PA and aldehyde group on oxidized and unoxidized PA could cross-link with the amino group on gelatin through dynamic Schiff base or Michael addition interactions (Fig. 1b). The dual-dynamic bonds in the network endow the adhesives with good injectability and self-healing properties both in mechanical and adhesive strength. Besides, GT could form helical bundles via hydrogen bond at room temperature (25 °C) while become random coils at higher temperature (37 °C). The high segment mobility of the adhesive at body temperature ensures the full contact between the sealant and the microsurface of tissue and increases the possibility of surface interaction, which could effectively enhance the adhesive strength (Fig. 1b–d). The temperature-dependent adhesive properties could avoid the inconvenience caused by the adhesion on instruments at room temperature. And it might take several minutes for the temperature of the gel to warm up to body temperature, during which the mispositioning hydrogel could be removed and repositioned (Fig. 1c, S1). More important, high segment mobility of the networks is conducive to the self-healing of the hydrogel, which makes secondary closure of reopened wounds possible. And the sealants could be repositioned several times to ensure the closure of reopened skin tissues without significant decrease in adhesive strength. Besides, the prepared adhesive sealants have good biocompatibility and biodegradability, photo-thermal antibacterial and hemostatic effect, and the capability to promote the closure and healing of the incisions. All those traits demonstrate that the developed adhesive hydrogel could serve as a versatile tissue sealant.

## Experimental Section

### Preparation and Characterization of the Adhesive Hydrogel

The adhesive hydrogel is composed of two precursor solution. Solution A is prepared according to a previous literature with some modification [35]. Briefly, solution A is composed of anhydrous ferric chloride (24 mg), Tris (500 mg) and PA (242.8 mg), which is mixed in 700 μL deionized water. And solution A is stirred for 3 h to ensure the formation of stable coordination bonds between PA and ferric ion (Fe (III)) and the polymerization of catechol group in PA, which is denoted as TPF. Moreover, solution B is composed of GT and SA. Briefly, GT (30%, w/v) is completely dissolved in deionized water in 80 °C, and then, SA (4%, w/v) is added into the gelatin solution and stirred until the fully dissolution of it. And the adhesive hydrogel is fabricated by the simple mix of solution A and B according to certain composition (Table S1), and the adhesive hydrogels are denoted as GT-SA-TPF_x_, in which x represents the content of TPF in the final adhesive hydrogel, and the final pH is around 8. Raman spectrum, Fourier transform infrared spectroscopy (FT-IR) and X-ray photoelectron spectroscopy (XPS) spectrum were recorded for the chemical characterization of the hydrogel. Moreover, rheological test, swelling test, degradation test, antioxidant efficiency evaluation, photothermal and near-infrared (NIR)-assisted photothermal antibacterial test, scanning electron microscope (SEM) and self-healing test were performed to further investigate the properties of the hydrogel. The details are available in Supporting Information.

### Biocompatibility Evaluation

Hemocompatibility, cytocompatibility and in vivo host response were evaluated to confirm the biocompatibility of the adhesives. The details are available in Supporting Information.

### Adhesive Evaluation

The adhesive strength of the hydrogels on fresh porcine skin tissues were evaluated through lap shear test. Besides, the temperature-dependent adhesive properties and the repeated closure efficiency of the hydrogels for the reopened incision were evaluated as well. The details are available in Supporting Information.

### Hemostasis Evaluation

The blood clotting capacity of the hydrogel was evaluated through a dynamic whole-blood-clotting test. And the burst pressure of the adhesive hydrogel was evaluated through a designed apparatus according to a previous literature [14]. The hemostatic capacity of the gel was evaluated through a femoral vein bleeding model. All the animal experiments were performed according to the guidelines established by the committee on animal research at Xi’an Jiaotong University. The details are available in Supporting Information.

### In vivo Wound Closure Evaluation

All the animal experiments were performed according to the guidelines established by the committee on animal research at Xi’an Jiaotong University. The wound closure effect was evaluated through a full-thickness skin incision model. And histological analysis was performed to further evaluate the healing effect of the closed incisions. The details are available in Supporting Information.

### Statistical Analysis

All the data were presented as mean ± standard deviation. And Student’s t-test was conducted for further analysis of the data using statistical product and service solutions (SPSS), in which* p* value < 0.05 was considered statistical significance.

## Results and Discussion

### Preparation and Chemical Characterization of the Adhesive Hydrogel

**Fig. 2:**
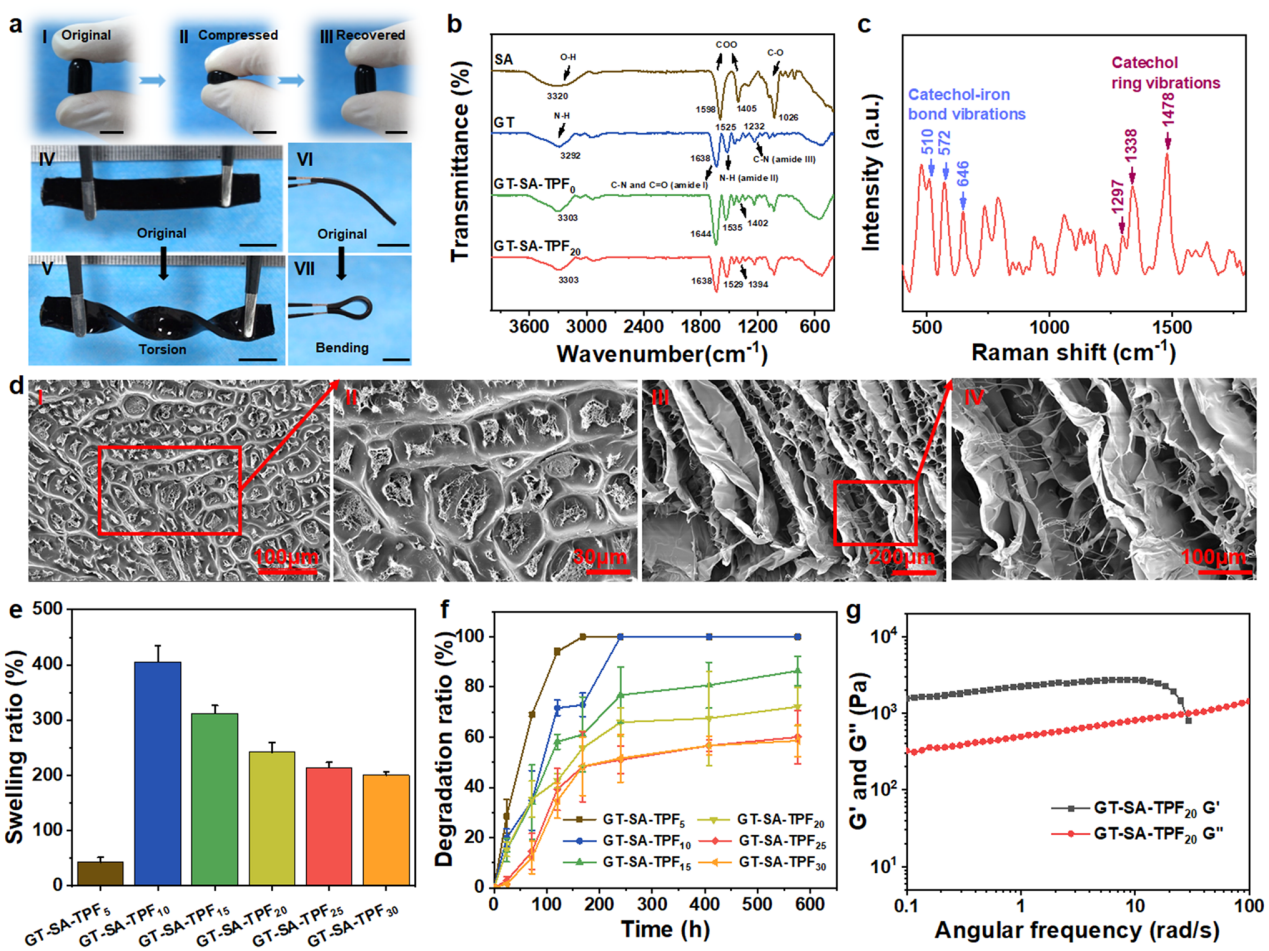
Characterization of the prepared hydrogels. **a** Original state (I), compressed state (II) and recovered state (III) of the hydrogel, and the original state (IV and VI) and distorted state (V and VII), scale bar: 1 cm. **b** FT-IR spectra of the samples. **c** The Raman spectrum of the GT-SA-TPF_20_ hydrogel sample. **d** SEM images of the lyophilized original (I and II) and swollen GT-SA-TPF_20_ hydrogel sample (III and IV). **e** The swelling ratio and **f** degradable behavior of the hydrogel samples. **g** Angular frequency sweep curve of GT-SA-TPF_20_ hydrogel samples

To fabricate bioinspired self-healing hydrogels with temperature-dependent adhesive properties, first, a compound system (TPF) composed of Tris, PA and Fe^3+^ has been prepared. Briefly, excess PA was added into the solution of Tris and Fe^3+^, catechol in PA and Fe^3+^ could form a stable tricomplex molecule (Fe(PA)_3_) through reversible coordinate bond under alkaline conditions (pH > 8.5) [3, 51], which has been proved in our previous work [3]. It has been declared that coordination with Fe^3+^ could prevent the autoxidation of catechol at high pH [52]. And the excessive amount of PA was oxidized and polymerized (Fig. S2). Next, TPF with different volume (x μL) was added into the mixture of GT and SA with equal volume to prepare the adhesive hydrogels (Fig. 1b), denoted as GT-SA-TPF_x_ (Table S1). The prepared hydrogel GT-SA-TPF_20_, as a model sample, could bear compression and distortion (Fig. 2a). The characteristic peaks at 1598 and 1405 cm^−1^ in the FT-IR spectrum corresponding to the asymmetric stretching vibration and symmetric stretching vibration of carboxyl group of SA (Fig. 2b), and the peak at 3320 and 1026 cm^−1^ corresponding to the O–H stretching and C-O stretching were observed. The FT-IR spectrum of GT exhibited characteristic absorption bands centered at 1638 cm^−1^ (amide I C=O and C-N stretching), 1525 cm^−1^ (amide II, N–H bending), 1232 cm^−1^ (amide III, C-N stretching) and 3292 cm^−1^ (N–H stretching) [53–56]. Compared with the FT-IR spectrum of SA, the characteristic absorption peaks of carboxyl group shifted from 1598 and 1405 cm^−1^ to 1644 and 1402 cm^−1^, and the absorption peak at 3320 cm^−1^ shifted to 3303 cm^−1^, which might be attributed to the new formed hydrogen bond between SA and GT [53–56]. Besides, the characteristic absorption peak at 1535 cm^−1^ in the FT-IR spectrum of GT-SA-TPF_0_ shifted to 1529 cm^−1^ and became less intensive in that of GT-SA-TPF_20_, which could be because the Schiff base reaction or Michael addition reaction occurred between the amino group of GT and PA (both unoxidized and oxidized) [57]. Meanwhile, it has been well demonstrated that Schiff base reaction or Michael addition reaction could occur among free aldehyde groups and oxidized catechol with amino groups of gelatin [19, 58–61]. Moreover, the absorption band centered at 1644 cm^−1^ shifted to lower wavenumber (1638 cm^−1^) and became broader and less intensive, which indicates the coordination of carboxyl group with Fe^3+^ [62–64]. The Raman spectrum depicted in Fig. 2c shows characteristic peaks centered at 500–700 cm^−1^ and 1200–1500 cm^−1^, which indicates the iron-catechol coordination [51, 52]. Besides, XPS test was carried out to further confirm the coordination of ferric ions. The deconvoluted O 1*s* signal of GT-SA-TPF_20_ sample showed two peaks centered at 533.12 and 531.89 eV, which is attributed to C–OH/Fe–OH groups and C=O/Fe–O groups (Fig. S3) [65, 66]. All those results demonstrated that the strong interaction between GT and SA (hydrogen bond), the Schiff base or Michael addition reaction between GT and PA and the coordination bond of Fe^3+^ and carboxyl or catechol group synergistically formed the bioinspired injectable hydrogel sealant.

### Morphology, Rheological Properties, Swelling Capacity and in vitro Degradability

The microstructure of the adhesive hydrogel was presented through SEM images (Fig. 2d). The original hydrogel sample showed dense structure. This could be attributed to the strong hydrogen bond formed between GT and SA inducing the collapse of the network during lyophilization. And the swelling hydrogel shows porous structure with microfibrils interwoven between the thin layers of the microstructure, mimicking the ultrastructure of byssal adhesive plaque [44], which is probably due to the self-assembly among GT and TPF mixture [67]. Besides, swelling behavior imposes significant influence on the mechanical properties of hydrogel materials, and the swelling ratio (SR) of the samples in PBS (1×) was investigated. As shown in Fig. 2e, the SR of the samples decreased from 405 to 200% as the amount of the TPF increased from 10 to 30 μL in certain hydrogel precursor solution, which demonstrates the increased bonding density in the hydrogel network. And it is noteworthy that the sample GT-SA-TPF_5_ shows the lowest SR than any other samples, which might could be attributed to the easier erosion of the hydrogel network with lower cross-linking density [3]. Moreover, degradation properties of adhesive hydrogels are essential for biomedical application, and the degradation rate of the samples is estimated as well. PBS (1×, pH 7.4) is applied to stimulate physiological environment. The results (Fig. 2f) are consistent with that of SR, indicating that the more TPF content, the slower degradation rate of the samples, which demonstrates the important role of TPF as cross-linking agent. Furthermore, rheological tests were also carried out to investigate the viscoelasticity of the adhesive hydrogels. The storage moduli of the final hydrogel (GT-SA-TPF_20_) are about 2400 Pa (Fig. S4). Storage moduli (*G*′) and loss moduli (*G*″) of GT-SA-TPF_20_ are increased as the angular frequency increasing in the range of 0.01–10 rad s^−1^ at 37 °C (Fig. 2g), demonstrating that the hydrogel is stable and behave like an elastic solid, and *G*′ of the hydrogel is consistently higher than *G*″ as the oscillation frequency increased (0.01–10 rad s^−1^), which indicates the dynamic hydrogel network [2, 14].

### Self-healing Behavior and Injectability of the Hydrogel

**Fig. 3:**
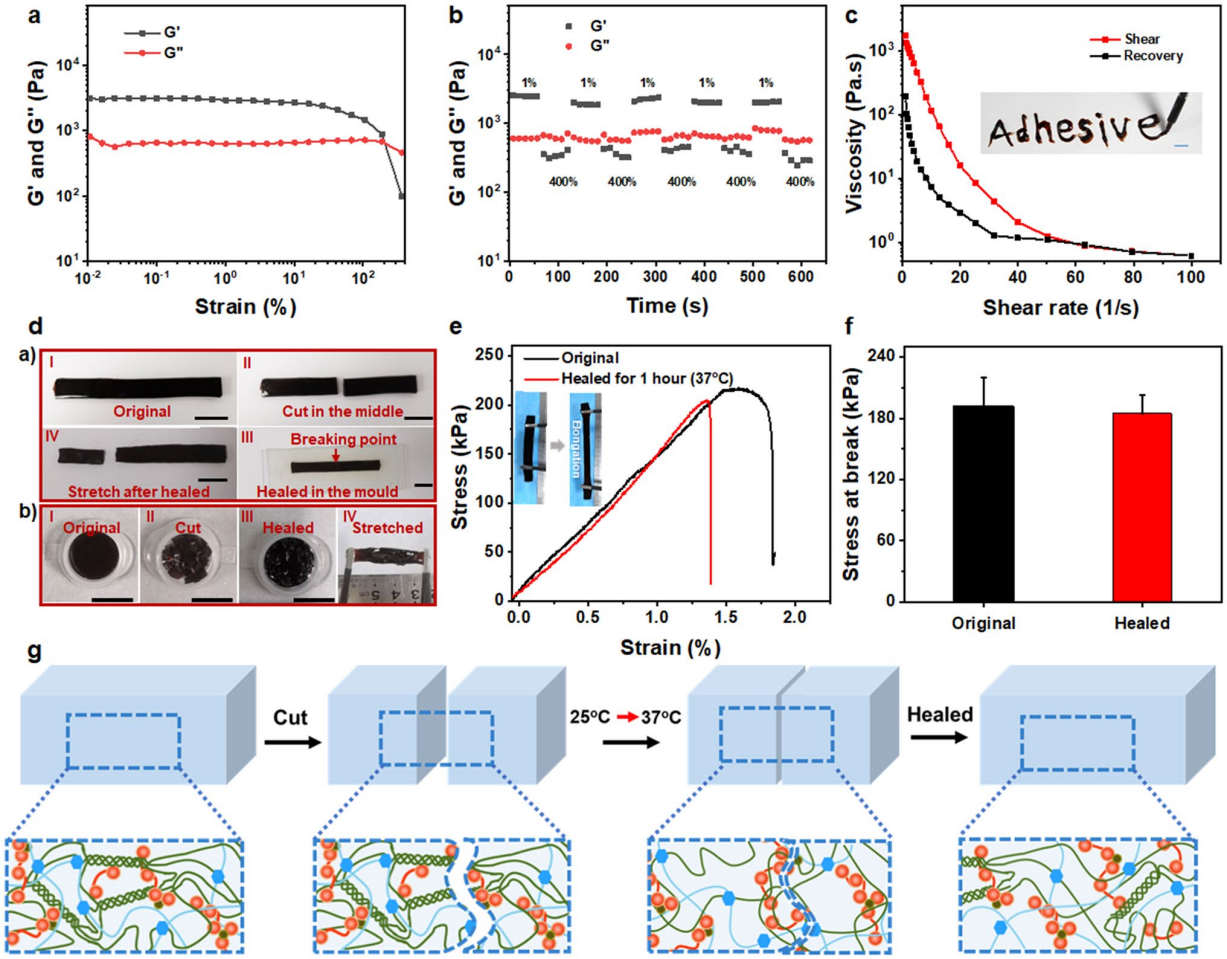
Self-healing properties and injectability of the adhesive hydrogel GT-SA-TPF_20_. **a** Strain sweep of the hydrogel within the strain range of 0–500%. **b** Rheological self-healing properties of the hydrogel with strain switched from 1 to 400% for 5 cycles. **c** Shear rate-dependent viscosity of the hydrogel. Inset: image showing the injectability of the hydrogel. **d** Macroscopic self-healing display, **a**) The original (I), fractured (II), healed (III) for 1 h and the secondary fractured (IV) state of the hydrogel, **b**) The original (I), shattered (II), healed (III) for 3 h and stretched (IV) state of the hydrogel. **e** Stress–strain curves and **f** fracture stress of the original hydrogel and healed sample after cured for 1 h at 37 °C. Inset: images showing the tensile property of the hydrogel. **g** Illustration for the self-healing mechanism of the adhesive hydrogel. Scale bar: 1 cm

Self-healing capacity of sealants is crucial for the repeated incision closure, and it could prolong the lifespan of materials in case of destroyed by external mechanical force. Therefore, the autonomous healing capacity of the adhesive hydrogel was estimated as well. To investigate the rheological self-healing properties, strain sweep was carried out to determine the linear viscoelastic region and critical point where the collapse of the hydrogel network occurred. As depicted in Fig. 3a, the moduli are consistently stable in the linear viscoelastic region (about 0.01–10%) and the value of *G*′ is higher than that of G″ with strain lower than 200%, indicating the good elasticity of the hydrogel [68]. *G*′ and *G*″ of the hydrogel decreased drastically once the strain exceeded the critical strain point (~ 200%). Next, the rheological recovery capacity of the adhesive hydrogel was evaluated through continuous alternative strain sweep with the strain shifted from 1 to 400% for 5 cycles (Fig. 3b). The value of *G*′ decreased from 2522 to 354 Pa while the strain switched from 1 to 400%, and the collapse of the network occurred (*G*′ < *G*″). The moduli of the GT-SA-TPF_20_ almost recovered to its original state (~ 2000 Pa) immediately once the strain shifted to 1%. The rapid and repeated recovery capacity during the alternative strain cycles demonstrated the excellent self-healing capacity. The shear-thinning results demonstrate that the viscosity of the hydrogels decreases as the shear rate increases, and the viscosity recovery shows an obvious hysteresis due to the dynamic cross-linked networks as the shear rate decreases from 100 to 0 rad s^−1^ (Fig. 3c). The shear-thinning properties enable the injectability of the adhesive hydrogel, and the self-healing capacity ensures the functionality of the network after injection.

Besides the rheological healing capacity, the macroscopic and mechanical self-healing properties at 37 °C are investigated as well. Strips of hydrogel sample were cut in the middle and healed at 37 °C for 1 h, and then, the healed samples were stretched to fracture. As shown in Fig. 3d, the breaking point was not at the original fracture position. Meanwhile, the hydrogel was cut into pieces and put into a mold, and the hydrogel could be stretched (with a strain of ~ 200%) after healed at 37 °C for 3 h. Moreover, the recovery of mechanical strength of the healed hydrogel was estimated through tensile test (Fig. 3e, f). It turned out that the stress–strain curves of the healed samples share similar profiles with that of the original sample, and the mechanical strength at break is almost the same as that of original sample. All those results indicate the excellent self-healing capacity of the sample. The rapid self-healing capacity of the hydrogels could be synergistically attributed to the dynamic coordination between carboxyl/catechol group and Fe^3+^, Schiff base reaction between aldehyde/quinone group and amino group of GT and the strong hydrogen bond between GT and SA (Fig. 3g). Meanwhile, the mobility of the network is increased at 37 °C due to the temperature-dependent phase transition behavior, which is conducive for the full contact of those reactive sites and could further enhance the self-healing capacity of the adhesive hydrogel.

### NIR-assisted Photothermal Antibacterial Activity and Antioxidation

**Fig. 4:**
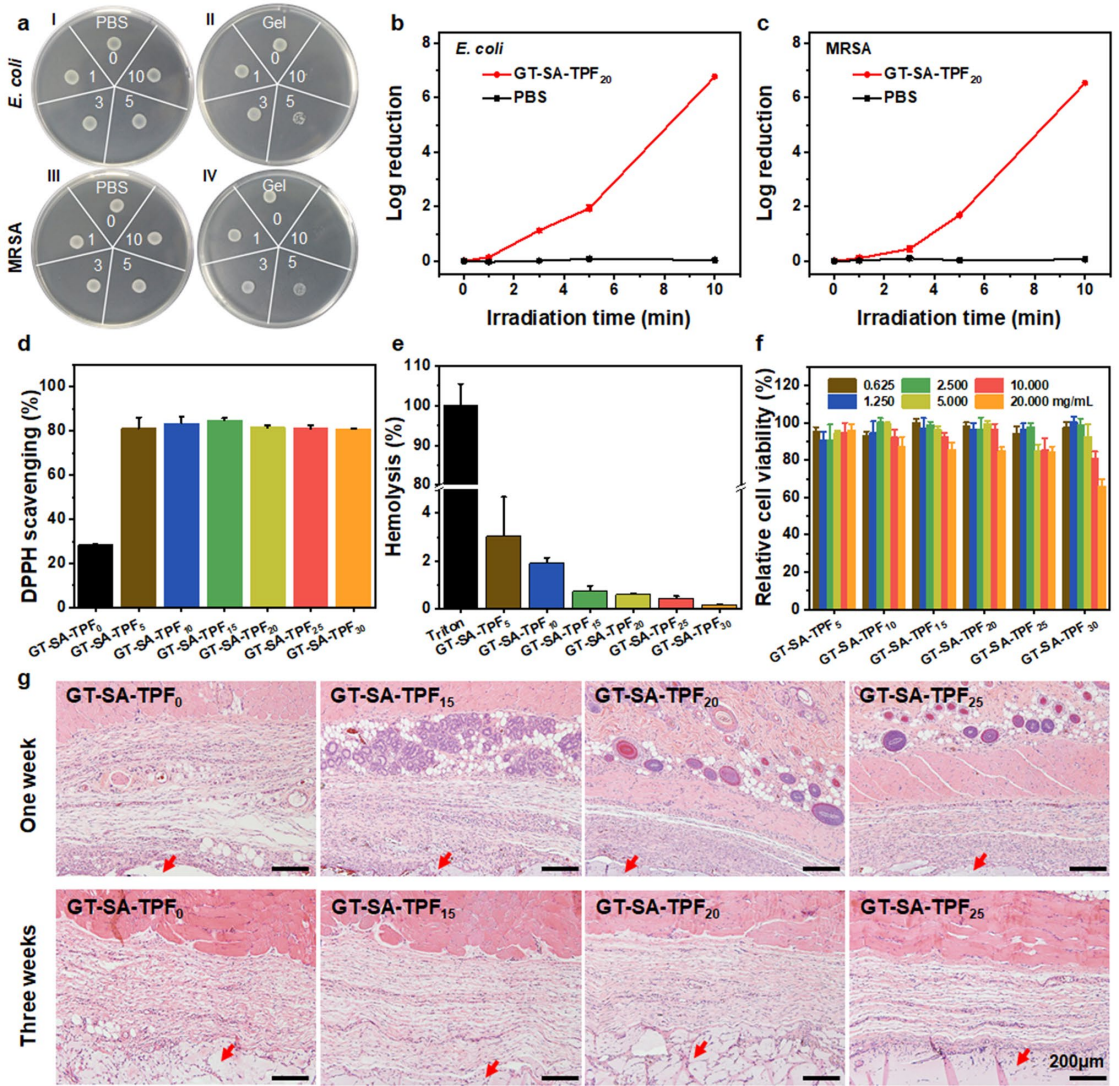
Antibacterial activity, antioxidation and biocompatibility of the hydrogels. **a** Images of the survival *E. coli* treated by PBS (I) and hydrogel (II) under NIR irradiation for different time. Images of the survival MRSA treated by PBS (III) and hydrogel (IV) under NIR irradiation for different time. The quantitative results of the antibacterial effect against **b** *E. coli* and **c** MRSA. **d** DPPH scavenging capacity and **e** hemocompatibility of the hydrogel samples. **f** Relative viability of L929 cells after incubated with leaching solutions of different samples at varied concentration. **g** H&E staining images of the skin tissues after subcutaneously implanted with different hydrogel samples for one and three weeks. Arrow: the site of the samples

Infection has been one of the major challenges for chronic wound healing, and photothermal-assisted antibacterial strategy has been adopted as an effective bactericidal method without resistance. The coordination of Fe^3+^ and catechol has been proved with absorption in near-infrared region and has good NIR-assisted photothermal effect. The temperature enhanced significantly (Δ*T* = 27.6 °C) for GT-SA-TPF_20_ during the NIR irradiation for 10 min, and the temperature enhancement for GT-SA-TPF_0_ is no more than 1 °C (Fig. S5), indicating the good photothermal effect of hydrogel GT-SA-TPF_20_. Meanwhile, the in vitro NIR-assisted photothermal antibacterial effect of GT-SA-TPF_20_ against *E. coli* and MRSA was investigated (Fig. 4a–c). The results turned out that almost all the bacteria were killed after irradiated for 10 min, which demonstrated the good antibacterial effect of the hydrogel against Gram-negative and drug-resistant bacteria.

Besides, excessive production of reactive oxygen species (ROS) in wound site might cause oxidative stress and cytotoxicity due to the damage of DNA and enzymes [3, 33]. Therefore, the integration of antioxidation to adhesive hydrogel could effectively scavenge the overproduced ROS species and improve metabolism at the wound site to accelerate the healing process. The coordination with Fe^3+^ could prevent the autoxidation of catechol at high pH, and we hypothesized that those unoxidized catechol group with excellent antioxidant activity endows the adhesives with good ROS scavenging capacity. The antioxidation of the hydrogels was estimated by the scavenging capacity of the samples for a stable free radical 1, 1-diphenyl-2-picrylhydrazyl (DPPH·). The results (Fig. 4d) showed that all the hydrogel samples with TPF added could achieve a scavenging ratio over 80%, and the samples consisted of pure GT and SA (GT-SA-TPF_0_) show a scavenging ratio of 28%, which indicates the effective DPPH· scavenging capacity of the gels. The scavenging capacity of GT-SA-TPF_0_ might originate from the interaction between DPPH· and free amino group in GT.

### Hemocompatibility and Cytocompatibility and in vivo Biocompatibility

Biocompatibility is of great importance for biomedical material, and hemocompatibility included. Hemolysis test was carried out to evaluate the hemocompatibility of the hydrogel (Fig. 4e), with Triton X-100 as a positive control group and PBS as a negative control group. After incubated those samples and erythrocyte at 37 °C for 1 h, the quantitative results show that the hemolysis ratio of GT-SA-TPF_10_, GT-SA-TPF_15_, GT-SA-TPF_20_, GT-SA-TPF_25_ and GT-SA-TPF_30_ is no more than 2%, and the hemolysis ratio of GT-SA-TPF_5_ is higher but no more than the permissible limit of 5% [69], indicating their good hemocompatibility.

Cytocompatibility is an essential factor for adhesives as tissue sealant for post-wound-closure care. The cell compatibility of the hydrogels was estimated through a leaching pattern test (Figs. 4f, S6), and murine fibroblast (L 929) cells were selected as model cells. After incubated with those leaching solutions of different samples at varied concentration for 1 day, the cell viability of L929 cells was measured through a microplate reader. The results showed that the cell viability of GT-SA-TPF_5_, GT-SA-TPF_10_, GT-SA-TPF_15_, GT-SA-TPF_20_ and GT-SA-TPF_25_ was higher than 85% in spite of the concentration of the leaching solution increased from 0.0625 to 20.000 mg mL^−1^, indicating the good cell compatibility of those samples. Although the cell viability of GT-SA-TPF_30_ was about 90% when treated with leaching solution at concentration lower than 2.5 mg mL^−1^, and the cell viability decreased as the concentration of the leaching solutions increased, demonstrating the cytotoxicity could not be ignored. Besides, the live/dead staining was performed to uncover the morphology of L929 cells after treated with leaching solutions of the samples at a concentration of 20.000 mg mL^−1^. The majority of L929 cells with spindle morphology were stained green (live cells), and an obviously decreased cell density demonstrated the cytotoxicity of GT-SA-TPF_30_ (Fig. S6), which is consistent with the results of cell viability. Moreover, the in vivo long-term biocompatibility of GT-SA-TPF_15_, GT-SA-TPF_20_ and GT-SA-TPF_25_ was evaluated though a subcutaneous implantation method (Fig. 4g). As both GT and SA were documented with good biocompatibility, GT-SA-TFP_0_ was set as control group. Hematoxylin and eosin (H&E) staining were performed to evaluate the host response of implanted site. The H&E staining images of skin tissues with samples implanted for one week showed obvious acute inflammatory responses, which is comparable to that of the control group (Fig. S7). Moreover, the inflammatory response decreased as the hydrogels degraded, and the cells grow around the samples. All those results revealed their good cytocompatibility and in vivo biocompatibility.

### Temperature-dependent Adhesive Capacity

**Fig. 5:**
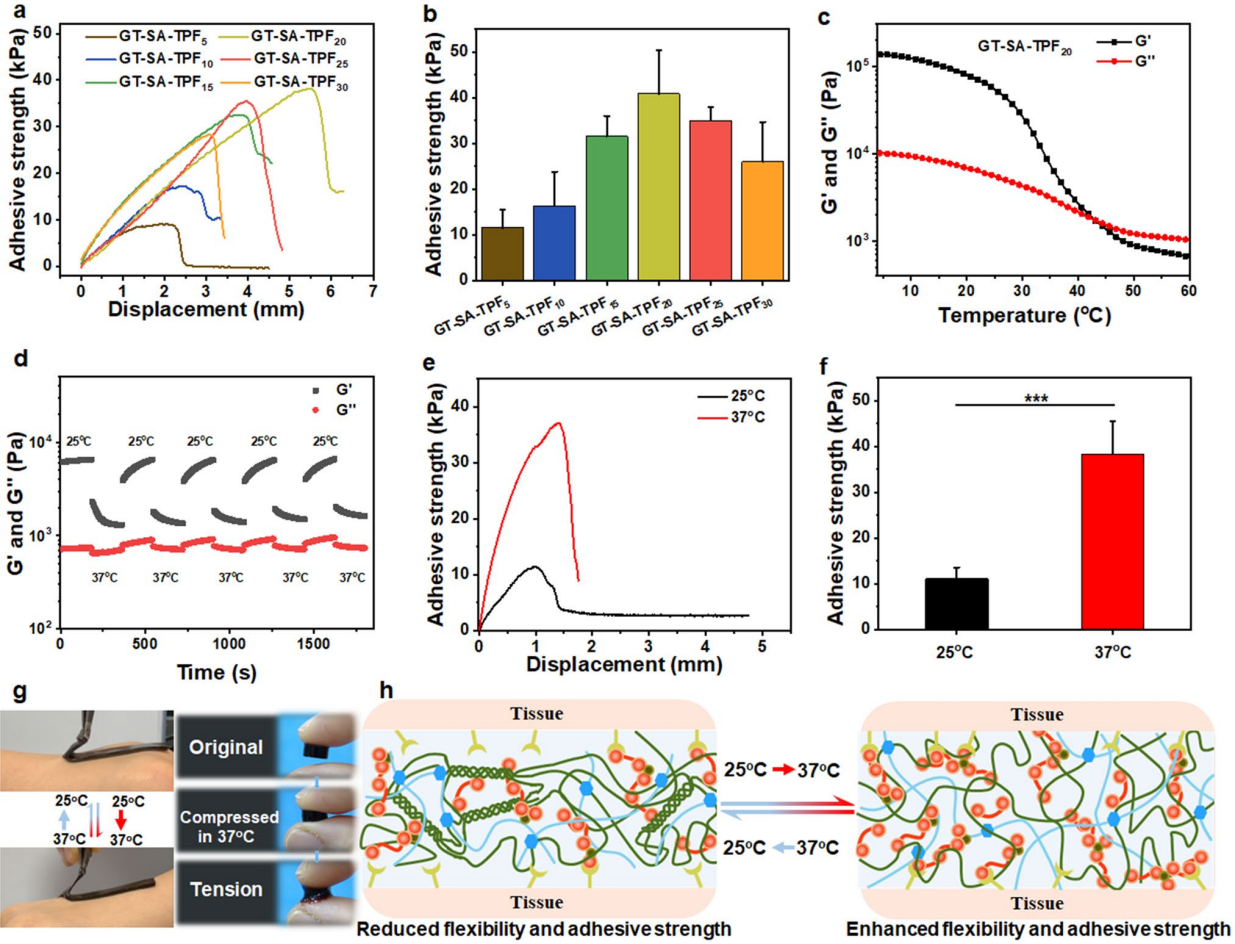
Adhesive capacity and temperature responsive adhesion of the hydrogels on porcine skin tissues evaluated through lap-shear test. **a** Adhesive strength-displacement curves and **b** adhesive strength of different hydrogel samples on porcine skin tissue. **c** The temperature-dependent moduli of the hydrogel GT-SA-TPF_20_. **d** Time sweep of the hydrogel GT-SA-TPF_20_ with temperature alternated from 25 to 37 °C for 5 cycles. **e** Adhesive strength-displacement curves and **f** adhesive strength of the hydrogel GT-SA-TPF_20_ on porcine skin tissues at different temperature. **g** The presentation of temperature-dependent adhesion on human skin tissues. **h** Illustration of the proposed temperature-dependent adhesion mechanism. ***p < 0.001

Adhesive property of the hydrogel is of vital importance for tissue sealant. The adhesive strength of those prepared hydrogel on fresh porcine skin tissues was estimated through a lap shear test (Fig. S8). As shown in Fig. 5b, the adhesive strength of GT-SA-TPF_5_, GT-SA-TPF_10_, GT-SA-TPF_15_, GT-SA-TPF_20_, GT-SA-TPF_25_ and GT-SA-TPF_30_ was 11, 16, 31, 40, 35, and 26 kPa, which indicates that the adhesive strength of the samples increased and then decreased as the amount of TPF increased. And the fracture displacement of the bonded tissues witnessed similar change tendency (Fig. 5a). This phenomenon could be ascribed to the increasing cohesion of the hydrogels as TPF increased. It is widely accepted that the adhesion strength is dependent on both cohesion and surface adhesion [40, 70]. The increased adhesion at the beginning might be attributed to the increased cohesion of the samples, in which cohesion is the restrictive factor for the achieving of strong adhesive strength. However, the adhesive strength might decrease as the amount of cross-linker increased due to the reduction in reactive site for surface adhesion. The stronger adhesive strength of GT-SA-TPF_20_ compared with other samples might originates from the relatively balance between cohesion and surface adhesion.

Besides, to avoid the inconvenience caused by the adhesion of the sealants on instruments during operation, responsive-adhesion of hydrogel is necessary. Temperature ramp was performed in the range of 4–60 °C to investigate the thermorheological property of GT-SA-TPF_20_. *G*′ and *G*″ of the hydrogel decreased as the temperature enhanced, and the samples behave like a gel (*G*′ > *G*″) as the temperature is below ~ 43 °C, which indicates that the hydrogen bond in GT and SA gradually decreased during the temperature enhancement (Fig. 5c). And the rapid and reversible moduli (*G*′ and *G*″) alternate occurred and almost recover to its original value as the temperature switched between 25 and 37 °C for consecutive 5 cycles (Fig. 5d), which demonstrates the stable thermo-responsiveness of GT-SA-TPF_20_.

**Fig. 6:**
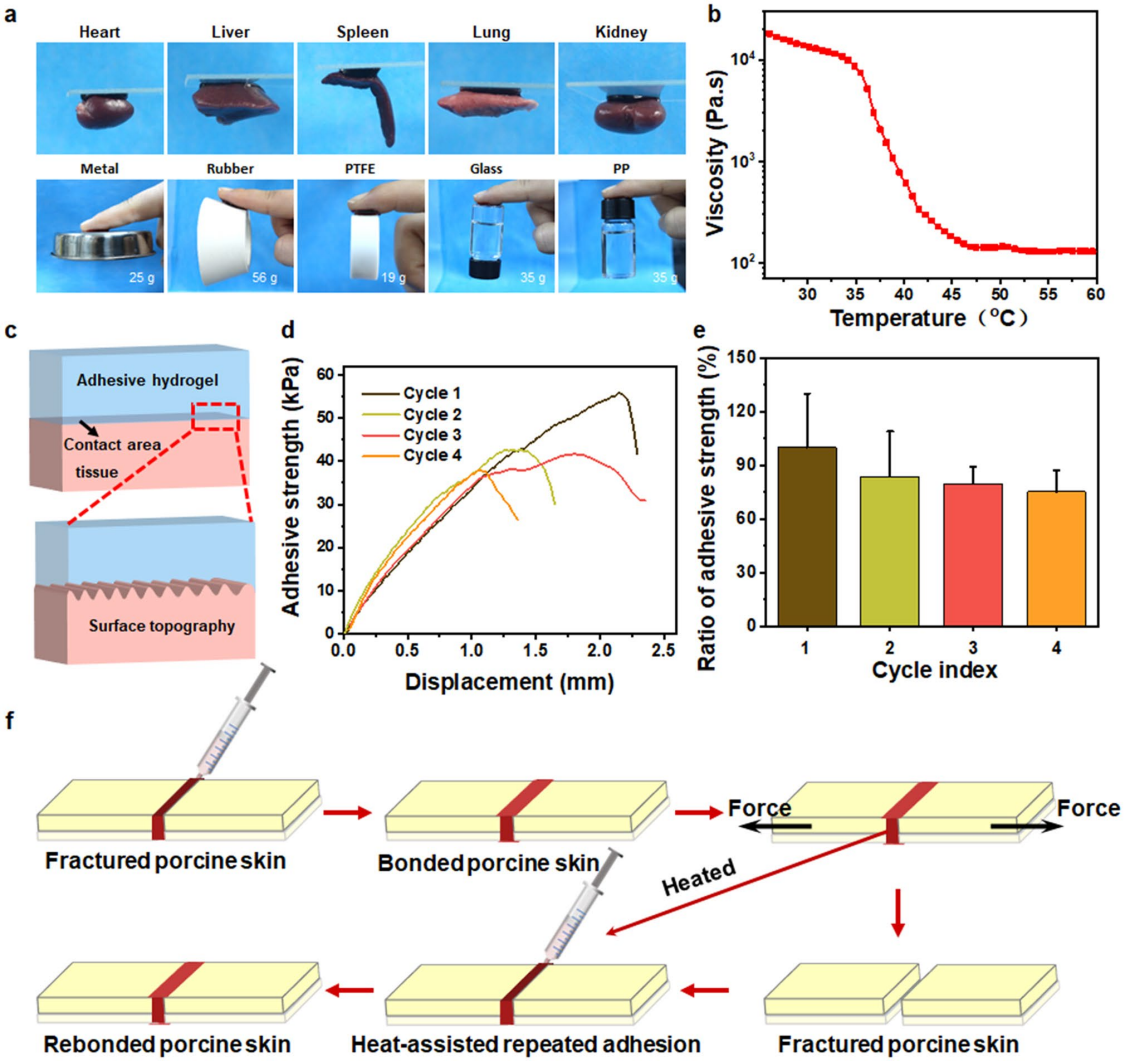
Adhesive properties of the hydrogel. **a** Presentations of the hydrogels adhered to different surfaces of the tissues or materials. **b** Temperature-dependent viscosity of hydrogel GT-SA-TPF_20_. **c** Illustration of the adhesive hydrogel in full contact with the surface of the tissues at body temperature. **d** Adhesive strength-displacement curves and **e** the ratio of adhesive strength of the repeated bonding cycles. **f** Illustration of the repeated temperature-responsive bonding of skin tissues

Painless removable is necessary when the mission of tissue sealant is over or when the mispositioning of adhesives occurred on dynamic tissue surfaces. Considering the stable thermo-responsiveness of GT-SA-TPF_20_, we envision that the temperature-responsive adhesion could be achieved through the control of the local temperature. As depicted in Fig. 5g, the hydrogel stored at 25 °C could be peeled easily immediately after positioned on the surface of human skin, while the hydrogel tightly adhered to the hands for a few minutes at 37 °C, indicating the temperature-dependent adhesive properties of the hydrogel. Furthermore, the temperature-responsive adhesive strength of the hydrogel on fresh porcine skin tissue was investigated through a lap shear test (Fig. 5e, f). The adhesive strength of the hydrogel improved significantly at 37 °C (38 kPa), which is obviously stronger than that at 25 °C (11 kPa). A proposed temperature-responsive adhesive mechanism is illustrated in Fig. 5h. The segment mobility of the GT is limited due to the formation of the helix bundles in network at room temperature, and the intramolecular hydrogen bond increased the cohesion of the adhesives, which confined the exposure of free amino group in GT and reduced the possibility of contact between the adhesives and the functional group including carboxyl (-COOH), hydroxyl (-OH), amino (-NH_2_) and thiol groups (-SH) [70]. And the adhesion failure is resulted from the poor surface adhesion (Fig. S1). Moreover, the drastically decreased viscosity of the hydrogel also demonstrated the decreased intermolecular interaction (Fig. 6b) and increased segment mobility as temperature enhanced [14, 71]. The hydrogels could adhere to the surface of the tissues through topological entanglement between the sealants and the micro-surface of the skin tissues due to the increased chain mobility at body temperature (Fig. 6c), which could increase the interactive site between the adhesion surfaces and enhance the adhesive strength. The thermo-responsive adhesive capacity is conducive to the detachment of the hydrogel through a local temperature change when necessary, which could also avoid the inconvenience caused by the adherent to other interfaces besides tissues. Except for the adhesion on skin tissues, the hydrogel showed adhesiveness to various substrates, including biological tissues (such as heart, liver, spleen, lung and kidney) and metal (25 g), rubber (56 g), Teflon (PTFE, 19 g), glass (35 g) and polypropylene (PP, 35 g), and the adhesive strength could support the weight of those materials (Fig. 6a).

Given the good self-healing capacity and temperature-responsive property of the hydrogel, we hypothesized that the hydrogel could achieve repeated closure of skin incision. As shown in Fig. 6f, two pieces of porcine skin tissues were bonded by the adhesive hydrogel, and the glued incision was pulled apart by external force. Then, the hydrogel adhered on the tissues was peeled off and placed at 45 °C for 5 min, after which the hydrogel was directly placed onto the fractured tissues. The results of tension tests indicated that the hydrogel could achieve repeated bonding of skin incision, and the value of bonding strength could reach almost 80% of the initial adhesive strength on the forth repeated bonding (Fig. 6d, e). All those results demonstrated that the hydrogel with responsive strong adhesion and repeated closure capacity shows promising application as tissue sealant.

### In vitro Blood Clotting Capacity and in vivo Hemostasis of the Hydrogel

**Fig. 7:**
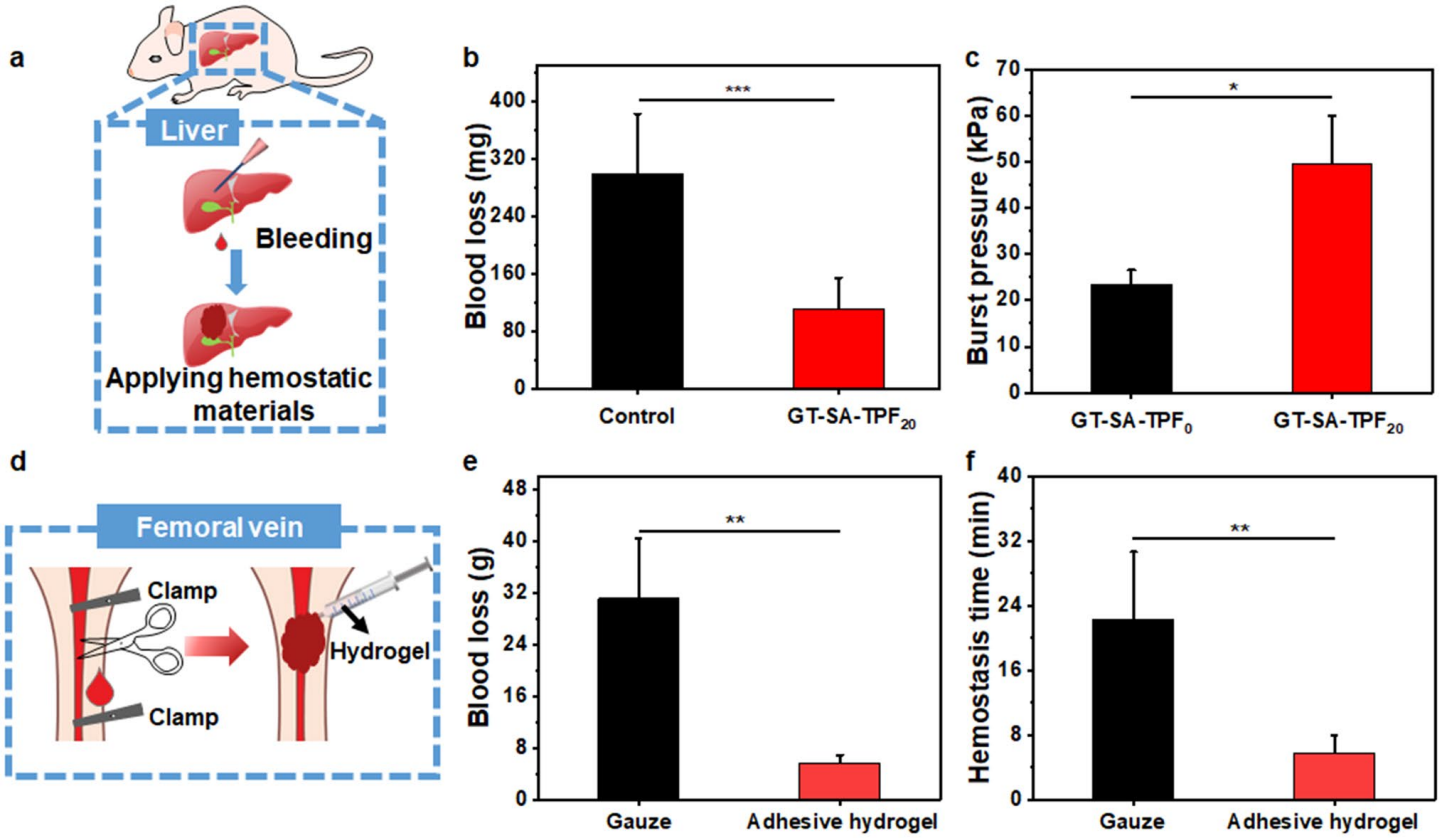
Hemostasis evaluation of the hydrogels. **a** Illustration for the creating superficial bleeding model and stopping bleeding by using the adhesive hydrogel in mouse liver. **b** Blood loss of the bleeding liver treated with different strategies. **c** Burst pressure of the samples. **d** Illustration for the creating acute bleeding model and stopping bleeding by using the adhesive hydrogel in rabbit femoral vein. **e** Blood loss and **f** hemostasis time of the bleeding femoral veins treated with different samples. *p < 0.05, **p < 0.01, ***p < 0.001

Given the good tissue adhesive capacity and biocompatibility, we envisioned that the adhesive hydrogel might be a promising candidate for surgical or accidental hemostasis. The blood clotting capacity of the hydrogels was estimated through dynamic whole-blood-clotting test at determined time, in which a lower BCI denotes a higher blood clotting rate [30]. The BCI of gauze, GT-SA-TPF_0_ and GT-SA-TPF_20_ is significantly lower than that of control group (Fig. S9), and the BCI of GT-SA-TPF_20_ is obviously lower than that of gauze and GT-SA-TPF_0_, indicating the good in vitro blood coagulation capacity due to the enhanced tissue adhesion of GT-SA-TPF_20_. Moreover, the in vivo hemostatic effect of the adhesive hydrogel was evaluated by using a hemorrhaging mouse liver bleeding model (Fig. 7a). As shown in Fig. 7b, the blood loss of the bleeding liver treated by adhesive hydrogel was significantly decreased (~ 110 mg) compared with that without treatment (~ 300 mg), and the hemostasis time was significantly reduced (Fig. S10), indicating the good hemostatic effect of the hydrogel. Besides superficial tissue bleeding, intraoperative or accidental blood vessel bleeding is also a challenging problem, in which blood pressure is an important factor that could not be ignored. A burst pressure test was performed to estimate the capacity of GT-SA-TPF_20_ as a sealant to withstand the pressure caused by blood or body fluids. After adhered to tissues to seal a 2 mm diameter hole, the tissue is fixed on the chamber (Fig. S11). Then, PBS was pumped into the chamber until the pressure is strong enough to break through the adhesives, and the maximum pressure during the process was recorded though a digital manometer. As shown in Fig. 7c, the burst pressure of GT-SA-TPF_20_ (~ 49 kPa) is obviously higher than that of GT-SA-TPF_0_ (~ 23 kPa), which is significantly higher than the normal blood pressure of human (10–16 kPa) [14, 19]. The results of the burst pressure test indicate the promising application of the adhesive hydrogel as sealant to prevent leakage of blood vessels. Therefore, the in vivo hemostatic capacity of GT-SA-TPF_20_ was evaluated through a rabbit femoral vein bleeding model (Fig. 7d). The blood loss and hemostasis time of the bleeding femoral veins treated with GT-SA-TPF_20_ is obviously less than that of the control group (Fig. 7e, f), which could be ascribed to a physical hemostasis barrier formed by the strong adhesion between the sealant and the femoral vein near the hemorrhage site. The results demonstrate the good hemostatic effect of the hydrogel in vivo and the applicability of the gels in complex wounds with irregular shapes.

### In vivo Wound Closure and Healing of the Hydrogel GT-SA-TPF_20_

**Fig. 8:**
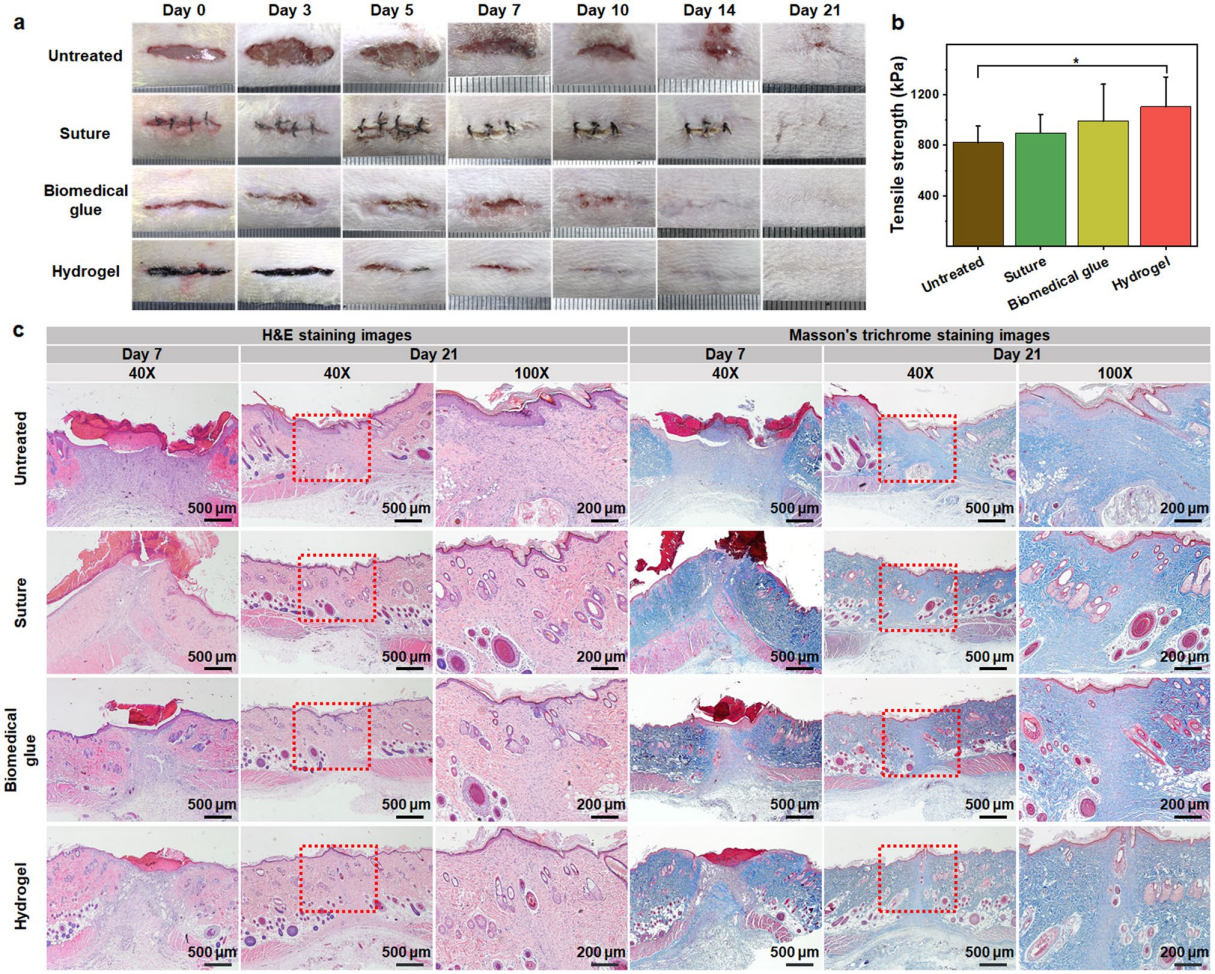
In vivo wound closure and healing evaluation. **a** Images of the incisions closed by suture, biomedical glue, adhesive hydrogel, and the wound without treatment was set as control. **b** The tensile strength of the healed skin tissues on day 21. **c** Images of H&E staining and Masson’s trichrome staining of the skin tissues after healed for 7 and 21 days. *p < 0.05

The results mentioned above demonstrated that the bioinspired adhesive hydrogels with excellent autonomous self-healing capacity, injectability, temperature-dependent adhesive property and good biocompatibility could be a promising candidate for sutureless post-wound-closure care. Given the strong adhesive capacity and good biocompatibility, GT-SA-TPF_20_ was selected for further investigation. The wound closure effect was evaluated through a full-thickness skin incision model. Four skin incisions (2 cm) were created on the back of the rat after anesthesia to reduce the influence of individual differences on the results. Next, those incisions were closed by suture, biomedical glue and adhesive hydrogels, and one without treatment was set as control group. The images of the incisions after healed for determined time are shown in Fig. 8a. The incisions treated by GT-SA-TPF_20_ show better closure than that treated by suture and biomedical glue, and the closure effect of those treatment is significantly better than that without any intervention. The wound became larger at the very beginning due to the movement of the rats, which delayed the healing process. Although the incisions are well closed by suture and biomedical glue, the scar caused by suture and the redness and certain damage caused by biomedical glue could not be ignored. On day 21, the incision closed with the adhesive hydrogel is almost healed, while obvious wounds still remain in the incisions without treatment or treated by suture. Although the incision treated by biomedical glue is closed better than that of suture and untreated, the regenerated tissues around the incision showed less hair compared with that treated with hydrogel. Moreover, after treated for three weeks, the incisions closed by different strategies were harvested and the strength of the healed skin tissues was evaluated through tension test. The results demonstrated that the stronger mechanical strength of the skin tissues treated by hydrogel, compared with other groups (Fig. 8b).

Besides, histological analysis was performed to further evaluate the healing effect of the closed incisions on day 7 and 21 (Fig. 8c). Consistent with the results depicted in Fig. 8a, the incisions treated by suture, biomedical glue and adhesive hydrogel show better closure, but the wound is smoother for biomedical glue and hydrogel closed incisions. The wounds treated with hydrogel show mild inflammatory response due to the exogenous materials. And more inflammatory response was observed in biomedical glue-treated wounds due to the poor biocompatibility, which is better than that of untreated group. Masson’s trichrome staining results demonstrated that more collagen deposition in treated three groups indicated better post-wound-closure care. Although all the treated incisions are almost completely closed on day 21, the wound healed by the biomedical glue showed inadequate epithelization and fewer skin appendages, especially for new hair follicles, compared with that treated by suture and adhesive hydrogel. Meanwhile, there is no residues of hydrogel, infections or serious inflammatory in the wound site treated by hydrogel, and fibroblasts at the bottom of the wound indicated no deep openings in the tissues. On day 21, the collagen fibers in all the treated wound site is denser than that on day 7, but the collagen fibers in the healed tissues treated by hydrogel are better arranged compared with other groups. Those results indicated that although the incision could be well closed by suture and biomedical glue with strong adhesive strength, the poor post-wound-closure healing could probably reduce the therapy compliance of patient, and the hydrogel with high adhesive strength and good biocompatibility could well close the incision and promote the healing of sealed wounds.

## Conclusions

In summary, the dynamic bonds cross-linked hydrogel with excellent self-healing capacity, injectability and shape adaptability, antibacterial activity, good biocompatibility and temperature responsive strong adhesive properties was facilely prepared for post-wound-closure care. The thermal-responsive phase transition of GT and the dynamic bonds in the network endows the hydrogel with good self-healing capacity, injectability and shape adaptability, and strong adhesive strength at body temperature. Moreover, the temperature-dependent adhesive capacity of the hydrogel equipped the sealant with operation convenience including fault-tolerant adhesion and undesired adherent on instrument, and makes it easier for the detachment of the hydrogel through local temperature control. Besides, the excellent self-healing capacity enables the repeated closure of reopened incision. Meanwhile, the results demonstrated that the adhesive hydrogel could effectively close the incision and promote the healing process. Accordingly, the adhesive hydrogel meets the requires for a versatile tissue sealant, including sufficient bonding strength for efficient wound closure, injectability and self-healing capacity with facile operation and no external stimuli, fault-tolerant adhesion and secondary closure of reopened wounds, biocompatibility and bioactivity for post-wound-closure care. All those desired traits make the hydrogel a promising candidate for sutureless wound closure and healing.

## Supplementary Information

Below is the link to the electronic supplementary material.

Supplementary file1 (DOCX 3268 kb)
