# Supplementary material for: Bioinspired Injectable Self-Healing Hydrogel Sealant with Fault-Tolerant and Repeated Thermo-Responsive Adhesion for Sutureless Post-Wound-Closure and Wound Healing
Source: Nanomicro Lett. 2022 Sep 13;14:185. doi: 10.1007/s40820-022-00928-z (PMC9470803; doi:10.1007/s40820-022-00928-z)
Supplement: Supplementary file 1 — Supplementary file1 (DOCX 3268 kb) [file 40820_2022_928_MOESM1_ESM.docx]

**Supporting information**

**Bioinspired Injectable Self-Healing Hydrogel Sealant with Fault-Tolerant and Repeated Thermo-Responsive Adhesion for Sutureless Post-Wound-Closure and Wound Healing**

Yuqing Liang ^a^, Huiru Xu ^a^, Zhenlong Li ^a^, Aodi Zhangji ^a^, Baolin Guo ^a, b*^

^a^ State Key Laboratory for Mechanical Behavior of Materials, and Frontier Institute of Science and Technology, Xi’an Jiaotong University, Xi’an, 710049, China

^b^ Key Laboratory of Shaanxi Province for Craniofacial Precision Medicine Research, College of Stomatology, Xi'an Jiaotong University, Xi'an, 710049, China

* To whom correspondence should be addressed. Tel: +86-29-83395340. Fax: +86-29-83395131. E-mail: baoling@mail.xjtu.edu.cn

**Methods and Experimental Section**

**1. Materials**

Gelatin (GT, ~300g bloom) is obtained from Sigma-Aldrich. Alginate sodium (SA) is purchased from Aladdin. Protocatechualdehyde (PA) is acquired from J&K Chemical. tris(hydroxymethyl) aminomethane (Tris) is obtained from Energy Chemical. Anhydrous ferric chloride is obtained from Alfa Aesar. All the materials and chemical reagents were used without further purification unless otherwise stated.

**2. Chemical characterization of the adhesive hydrogel**

To confirm the formation of the coordinate bonds between PA and ferric ion (Fe (III)), the surface of the lyophilized adhesive hydrogel was applied to collect Raman spectrum (Horiba, LabRAM Aramis spectrometer with confocal microscope) with a 785 nm laser line with the spectrometer pinhole. And the spectrum is collected within the range between 400 and 2000 cm^-1^. Besides, to further investigate the chemical composition of the hydrogels, the lyophilized adhesive hydrogel GT-SA-TPF_20_, GT-SA-TPF_0_, GT and SA were compressed into a plate, and the total reflection FT-IR spectra of those sample were measured in the range of 4000–400 cm^−1^ by employing a Nicolet 6700 FT-IR spectrometer (Thermo Scientific Instrument). And the XPS spectrum is collected by employing a spectrometer (Thermo Fisher ESCALAB Xi^+^) with an Al Kα X-ray source (1486.6 eV). The spectrum was processed through Avantage software.

**3. Rheological test**

The rheological test of the hydrogels was carried out by employing a TA rheometer (DHR-2). 500 μL hydrogel precursor solution was placed on the temperature control platform, and then the flat-panel detector with a dimeter of 20 mm approached the temperature control platform according to certain program until the gap is 1000 μm. Unless other stated, the frequency and strain were set as 10 rad/s and 1%, respectively, and the test temperature is set at 37^o^C. Moreover, to avoid water evaporation, the samples were sealed with silicone oil. Briefly, the strain sweep test was performed to uncover the linear viscoelastic region and the fracture strain of the adhesive hydrogel in the range of 0.01% ~ 500%. The angular frequency sweep test was conducted in the range of 0.1~100 rad/s. The time sweep test was carried out to evaluate the storage and loss modulus. The rheological self-healing property of the hydrogel was evaluated through alternate strain sweep test with the strain switched from a small strain (1%, 60 s) to a large strain (400%, 60 s) for 5 cycles. The shear-thinning property of the adhesive hydrogel was evaluated to uncover the influence of shear rate to the viscosity of the hydrogel. The viscosity of the hydrogel was tested while the shear rate varied from 0 to 100 s^-1^ and varied from 100 to 0 s^-1^ immediately. The temperature sweep test was performed in the range of 4 ~ 60^o^C. The temperature-viscosity curve was recorded in the range of 4 ~ 60^o^C, and the temperature-dependent viscosity of the hydrogel was recorded while the temperature switched from 25 to 37^o^C.

**4. In vitro swelling test**

In vitro swelling test was carried out to determine the stability and swelling ratio of the prepared hydrogel. Briefly, the prepared hydrogel (200 μL) was immersed into 2 mL PBS (0.01 M pH 7.4) in PE tubes at 37^o^C, and the samples were taken out at determined time from the tube and weighed after the removal of the superficial water. The test is finished until the weight of the samples stops increasing. The swelling ratio (SR) of the samples is calculated by the following formula: SR= (W_t_ –W_0_)/W_0_ × 100%, in which W_0_ is the original weight of the hydrogel, and W_t_ is the weight of the samples at determined time.

**5. In vitro degradation test**

To investigate the in vitro degradation behavior of the hydrogels, the lyophilized samples (~100 mg, n = 3) were immersed into PBS (3 mL) at 37^o^C (shaking at 100 rpm). At determined time, the samples were taken out from the PBS and rinsed with DI water to remove excess salinity. The samples were weighed after drying, and the degradation rate (DR) was calculated by the following formula: DR = (W_0_ – W_t_)/W_0_ × 100%, in which W_0_ is the original weight of the hydrogels and W_t_ is the weight of the samples at determined time.

**6. Cytocompatibility test**

The cytocompatibility of the adhesive hydrogel was evaluated through a leaching pattern test. Briefly, the lyophilized adhesive hydrogels, GT-SA-TPF_x_ (x = 5, 10, 15, 20, 25, 30) were sterilized at 55°C for at least 24 h. The dulbecco’s modified eagle medium (DMEM) (Gibco) containing 10% fetal bovine serum (FBS) (Gibco), 1.0 × 10^5^ U/L penicillin (Hyclone) and 100 mg/L streptomycin (Hyclone) was used as the complete growth medium. The sterilized samples (20 mg/mL) were immersed into complete growth medium without FBS for 24 hours at 37^o^C, and the leaching solution was prepared after the centrifugation and filtration to remove the solid substances and bacteria. L929 cells were seeded in 96-well plate at a density of 10000 cells/well. After the adherence of the cells, the culture media was replaced with different concentrations of leaching solution with FBS added. The cell viability and morphology was uncovered by alamarBlue® assay and LIVE/DEAD® Viability/Cytotoxicity Kit assay, respectively. After incubation for 24 hours, the leaching solution was replaced with complete culture medium with alamarBlue® reagent added (10%, v/v). After incubation for another 4 hours in a humidified incubator containing 5% CO_2_ at 37 °C, 100 μL of the culture medium with alamarBlue® reagent added in each well was transferred into a 96-well black plate (Costar), and the fluorescence (560 nm as the excitation wavelength and 600 nm as the emission wavelength) was recorded by a microplate reader (Molecular Devices). Moreover, cell seeded in tissue culture plate (TCP) without intervention was set as control group, and tests were repeated five times for each group. For the observation of the cell morphology, after incubation with leaching solution with FBS added for 24 hours, the culture media was replaced with complete culture medium (no FBS), with LIVE/DEAD® reagent added according to the instruction book. After incubated for another 30 min, the culture medium was removed gently and rinsed with sterilized PBS for three times. Finally, the cell morphology was uncovered on an inverted fluorescence microscope (IX53, Olympus).

**7. In vivo biocompatibility evaluation**

All the animal experiments were performed according to the guidelines established by the committee on animal research at Xi’an Jiaotong University. Rats (female, Sprague Dawley, ~200 g) were anesthetized by injecting 10% chloral hydrate, and then 150 μL hydrogel samples were implanted subcutaneously under the skin tissue of the rats. Each rat was implanted with four different hydrogels samples, including GT-SA-TPF_0_, GT-SA-TPF_15_, GT-SA-TPF_20_, and GT-SA-TPF_25_. As GT and SA were widely accepted with good biocompatibility, GT-SA-TPF_0_ was selected as control group. After implanted for 7 and 21 days, the rats were sacrificed and the skin tissues with hydrogel implanted were harvest, which was embedded in paraffin, sectioned and mounted onto slides. And then hematoxylin and eosin (H&E) staining was performed to investigate the inflammatory response. The stained tissues were observed and analyzed by microscope (n = 3).

**8. Antioxidant efficiency evaluation**

A stable 1, 1-diphenyl-2-picrylhydrazyl (DPPH•) free radical was selected to evaluate the antioxidant efficiency of hydrogels. Briefly, 100 μL hydrogels were pulverized by a tissue grinder in ethanol (2.7 mL), and then 300 μL DPPH• (1 mM) in ethanol was added. And then the mixture was placed in a constant temperature shaker (37^o^C, 100 rpm) in dark. After half an hour, the mixture was centrifuged and the absorbance of supernatant was recorded by a UV–Vis spectrophotometer (n = 3). The DPPH• scavenging was calculated by the following formula: DPPH• scavenging = (A_b_ –A_h_)/A_b_ × 100%, in which A_b_ is the absorption of blank group (DPPH• in ethanol) and A_h_ is the absorption of experimental group (DPPH• in ethanol treated with different samples).

**9. In vitro hemocompatibility evaluation**

Mouse blood, with heparin sodium added, was centrifuged (1000 rpm) for 10 min to concentrate erythrocytes, with PBS (1x) as the medium. And the erythrocytes were washed with PBS for three times. The pure erythrocytes were diluted with PBS with a final concentration of 5% (v/v). Hydrogels (500 μL) were added into 24-well microplate, and the wells replenished with PBS and 1‰ Triton x-100 were set as negative and positive control groups, respectively, and then 500 μL erythrocytes solution (5%) was added to the wells with samples. After incubation at 37^o^C for 1 hour, the erythrocytes on the samples were dispersed and the solutions were centrifuged (1000 rpm) for 10 min. Next, the supernatant (100 μL) was added into a 96-well plate, and the absorbance at 540 nm was recorded by a microplate reader (Molecular Devices). The hemolysis ratio (%) was calculated by the following formula: hemolysis ratio (%) = = [(A_h_-A_l_-A_b_)/(A_t_-A_b_)] × 100%, in which A_h_ is the absorbance of the supernatant treated with hydrogel samples, A_l_ is the absorbance of leaching solution of the hydrogels, A_b_ is the absorbance of supernatant treated with PBS, and A_t_ is the absorbance of the supernatant treated with Triton. And the image of the hemolysis results is captured.

**10. Photothermal and photothermal-assisted antibacterial effect evaluation**

The photothermal effect of the prepared hydrogels was evaluated according to a previous literature [1]. Briefly, hydrogel samples (200 μL) were irradiated with near-infrared (NIR) light (1.0 W/cm^2^), and the temperature enhancement was recorded through a thermal infrared imager.

The NIR-assisted antibacterial effect against *Escherichia coli* (*E. coli*) and methicillin-resistant *Staphylococcus aureus* (MRSA) was investigated. Briefly, the sterilized hydrogels were placed into 48-well plate, and the 10 μL bacterial suspension in sterilized PBS (10^8^ CFU/mL) was added onto the surface of the samples. Then the bacteria on the samples were irradiated with NIR light (1.0 W/cm^2^) for 0, 1, 3, 5, 10 min, respectively. During the irradiation, the contact time between bacteria and hydrogel is controlled at 10 min. After irradiation, the bacteria were dispersed with PBS and seeded onto agar plate to allow the proliferation of the living bacteria. The colony-forming units were counted after cultured for 18 to 24 h at 37 °C. 10 μL of bacterial suspension (10^8^ CFU/mL) in PBS with different irradiation time was set as negative control group. The tests were repeated for three times for each groups. The antibacterial efficiency (%) was expressed as log 10 CFU according to the following formula: Log Reduction=Log cell count of control - Log survivor count on hydrogel.

**11. Scanning electron microscope (SEM)**

To uncover the morphology of the adhesive hydrogel, after sprayed the lyophilized samples with a thin gold layer, the scanning electron microscope (SEM) images of the original and swollen adhesive hydrogels were acquired by a field emission scanning electron microscope (FESEM; QUTAN FEG 250, FEI).

**12. Self-healing properties evaluation**

Rheological recovery test and macroscopic autonomous healing test were conducted to investigate the self-healing behavior of the hydrogels. The rheological recovery of the samples was uncovered through an alternative strain sweep test as mentioned in the rheological test section. As for the macroscopic autonomous healing test, the hydrogels were made into strips and cut in the middle, and then the two pieces of samples were put into the mold to ensure the contact of the broken parts. After placed at 37^o^C for 1 hour, the healing efficiency of the hydrogel was evaluated through a tension test. Besides, a circular hydrogel sample was cut into pieces and placed into the mold at 37^o^C for 3 hours, and then the healed sample was stretched.

**13. Adhesive capacity evaluation**

The adhesive capacity of the hydrogels on fresh porcine skin tissues were evaluated according to a previous literature [2, 3]. Briefly, porcine skin tissues were purchased from the market and scrape off the excess fat. The processed skin tissues were cut into rectangle (1 x 2 cm). 200 μL hydrogel precursor with different composition were added onto the surface of the porcine skin, and then another piece of skin tissue was placed onto the hydrogel precursor and press it gently. Next, the porcine skin tissues with hydrogel were placed at 25 and 37^o^C for 3 hours, respectively. The adhesive capacity of the hydrogel on porcine skin tissues were evaluated by using a lap shear test on a Materials Test system (MTS Criterion 43, MTS Criterion) equipped with a 50 N load cell at a rate of 2 mm/min. All these tests were employed more than 5 times.

**14. Repeated bonding capacity evaluation**

The adhesive capacity of the hydrogels on fresh porcine skin tissues were evaluated according to a previous literature with some modification [4]. Briefly, porcine skin tissues were purchased from the market and scrape off the excess fat. The processed skin tissues were cut into rectangle (2 x 2 cm). 200 μL hydrogel precursor with different composition was added onto the edge of the skin tissues, and the bonding strength was evaluated through tension test after placed about half an hour at 37^o^C. And for the following three times repeated bonding strength evaluation, the hydrogel was placed at 45^o^C for 5 min, and then the gels were placed onto the edge of the porcine skin tissue again. And the bonding strength was evaluated by the same method almost immediately.

**15. Burst pressure evaluation**

To evaluate the capacity of the adhesive hydrogel as tissue sealant, the burst pressure test was carried out through a designed apparatus according to a previous literature with some modification [5]. Briefly, fresh porcine skin tissues were cut into rectangle (2 x 2 cm), with a 2 mm-diameter hole created in the center of it. Then the skin tissue was fixed on a hole (diameter: 5 mm) in the upper surface of a custom-made cubic box, and ~ 200 μL hydrogel precursor was added onto the center of the skin tissue and placed at 37^o^C for 30 min to allow the crosslinking of the network. A digital pressure gauge (MIK-Y290, Asmik, China) and a syringe pump (20 mL) was connected onto the cubic box, and the cubic box and the tubes were filled with PBS before test. During the test, PBS was injected into the apparatus gently and the maximum burst pressure was recorded. The test was repeated for three times.

**16. In vitro blood clotting index evaluation**

The blood clotting index test was performed according to a previous literature with some modifications [6]. Briefly, calcium chloride (0.01 mol/L) contained citrated blood (50 μL) was added onto the surface of hydrogel samples (200 μL) or cottons in the tubes, which is incubated at 37^o^C for 2 min. And then deionized water with equal volume was added into the tubes to destroy the blood cells. The group of citrated blood with calcium chloride placed directly on the bottom of the tube (treated as mentioned) is set as control. And the absorbance of the solutions at 540 nm is recorded by a microplate reader (Molecular Devices). The blood clotting index was calculated by the following formula: blood clotting index = (A_s_ – A_b_)/(A_c_-A_b_) × 100%, in which A_s_ is the absorbance of blood solution treated with samples, A_c_ is the absorbance of blood solution in control group, and A_b_ is the absorbance of blank value. The test is repeated for three times.

**17. Hemostasis evaluation**

All the animal experiments were performed according to the guidelines established by the committee on animal research at Xi’an Jiaotong University. A hemorrhaging mouse liver bleeding model (Kunming mice, ~30 g, female) was applied to evaluate the superficial hemostatic effect of the gels. The liver of the mouse was exposed through abdominal incision after anesthetized, and pre-weighted filter papers on a paraffin film were placed beneath the liver tissue. Then a syringe needle (18G) was used to create the bleeding model in the surface of the liver, after which the hydrogels was positioned onto the bleeding site. The weight of the filter papers was weighed until the bleeding is stopped. Besides, New Zealand rabbits (~5 kg) were anesthetized by injecting 10% chloral hydrate. After the femoral vein was exposed, incisions were made on the vein with surgical scissors and intravenous clips were applied to stop bleeding, then 500 μL hydrogel precursor was injected around the incision. After ~5 min, the clips were removed. The blood loss during the process was dipped with pre-weighted gauze. The incisions on the veins treated with gauze and press gently is set as a control group. Finally, the weight of the gauze absorbed with blood is measured.

**18. In vivo wound closure evaluation**

The wound closure effect of the adhesive hydrogels was evaluated according to a previous literature with some modification [1]. All the animal experiments were performed according to the guidelines established by the committee on animal research at Xi’an Jiaotong University. Rats (~200 g) were fed for one week to acclimatize before experiment. The rats were anesthetized by injecting 10% chloral hydrate and their backs were shaved and sterilized with iodine. Then four incisions (2 cm in length) were made on the back of the rats, and the incisions were sealed by GT-SA-TPF_20_, and incisions sealed with suture, biomedical glue (Fuaile medical glue, consist of α-cyanoacrylate and n-octyl-α-cyanoacrylate) and no treatment were set as three control groups. Each group was repeated six times. The images of the wound were captured on day 0, 3^rd^, 5^th^, 7^th^, 10^th^, 14^th^ and 21^st^ day. The sealed skin tissues were harvested at 7^th^ and 21^st^ day, which is fixed with 4% paraformaldehyde for 1 h, embedded in paraffin, and then cross sectioned to 4 μm thickness slices. The obtained slices were then stained by Hematoxylin-Eosin (Beyotime, China) and Masson trichrome.

**Results**

Table S1: The composition of the hydrogel samples

| Sample | Solution A (μL) | Solution B (μL) |
| --- | --- | --- |
| GT-SA-TPF_5_ | 5 | 500 |
| GT-SA-TPF_10_ | 10 | 500 |
| GT-SA-TPF_15_ | 15 | 500 |
| GT-SA-TPF_20_ | 20 | 500 |
| GT-SA-TPF_25_ | 25 | 500 |
| GT-SA-TPF_30_ | 30 | 500 |


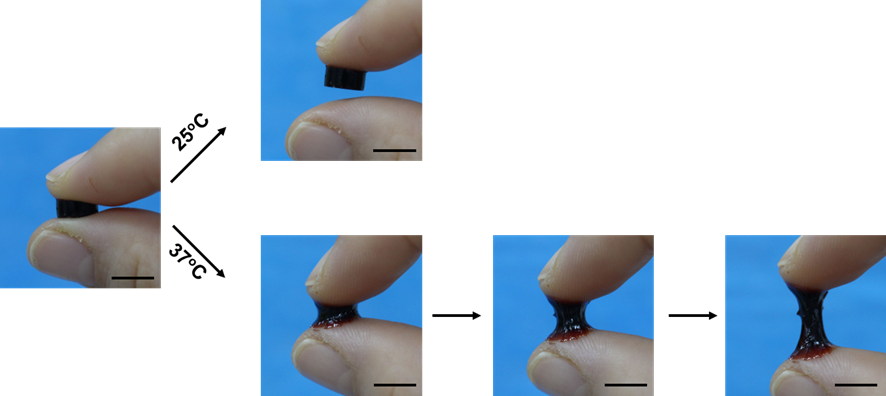


**Figure S1.** Presentation of temperature-dependent adhesive properties of the hydrogel. Scale bar: 1 cm.


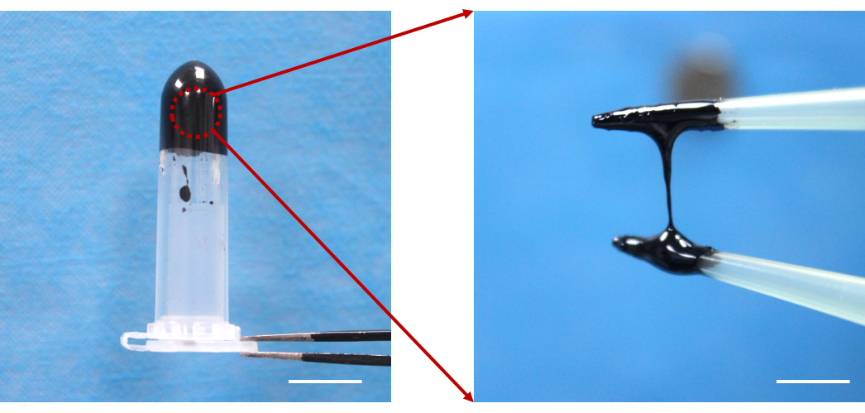


**Figure S2.** Presentation of the vacuum-dried TPF samples. Scale bar: 1 cm.


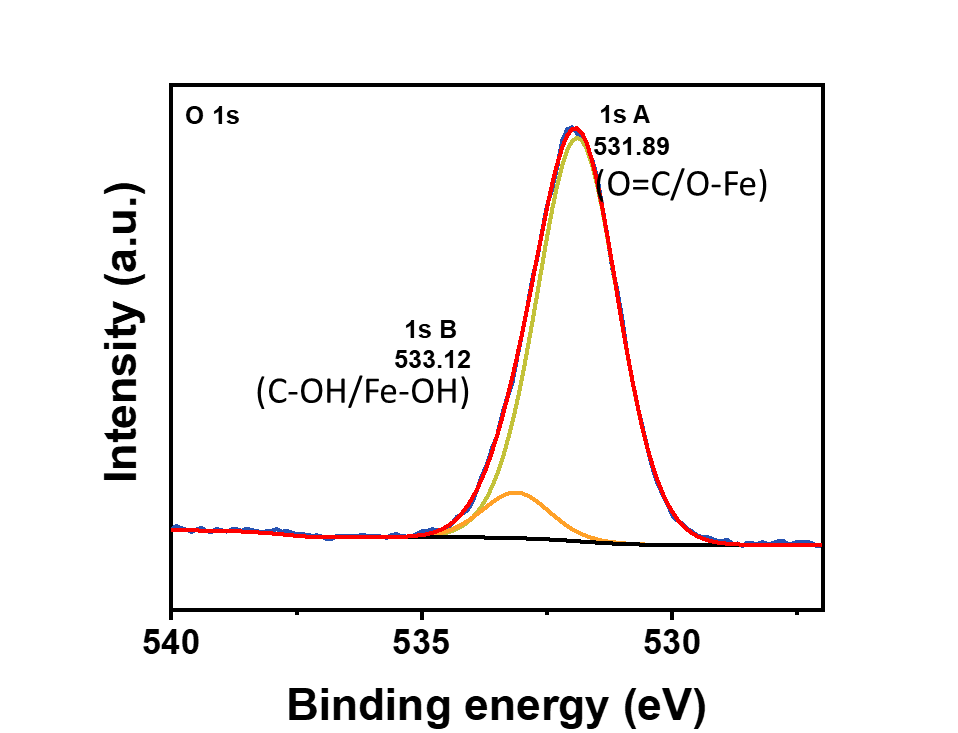


**Figure S3.** XPS spectrum of O 1s of the lyophilized GT-SA-TPF_20_.


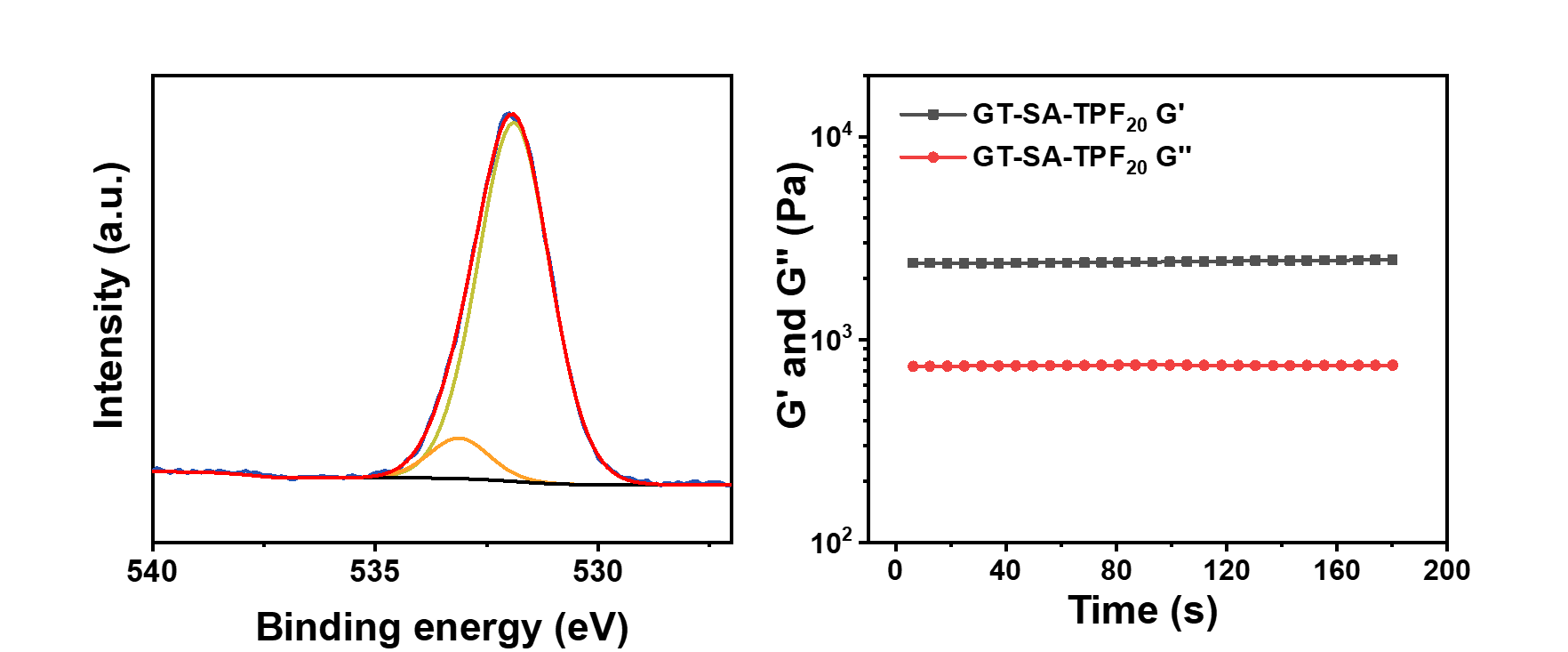


**Figure S4.** Time sweep of GT-SA-TPF_20_ hydrogel.


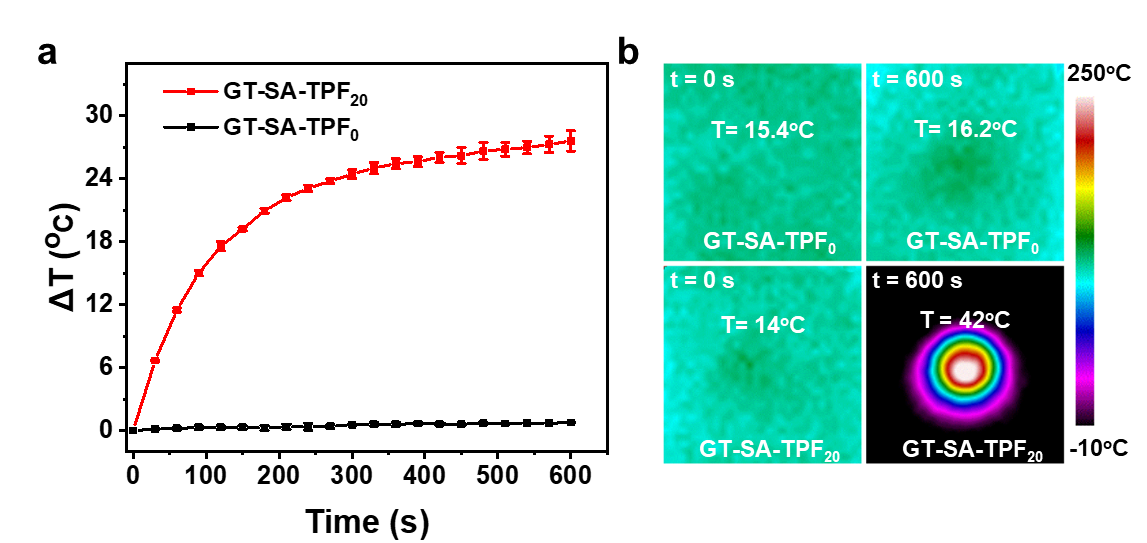


**Figure S5.** Photothermal effect of the hydrogels. **a** Temperature enhancement and **b** infrared thermal images of the samples under irradiation of NIR light (power density: 1 W/cm^2^).


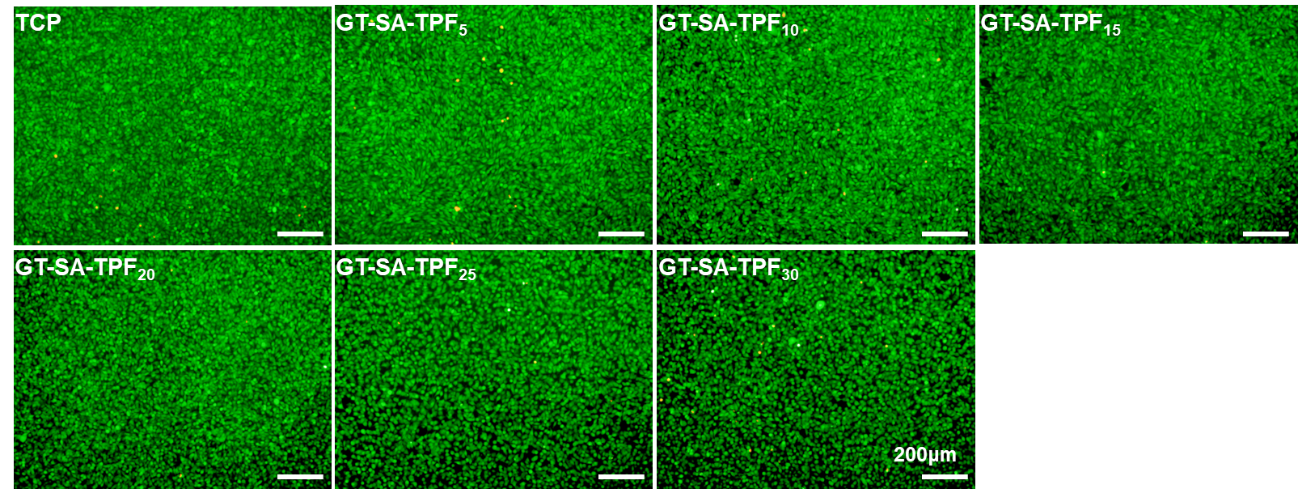


**Figure S6.** Live/dead staining images of L929 cells after incubated in leaching solutions of different samples with concentration at 20 mg/mL.

**
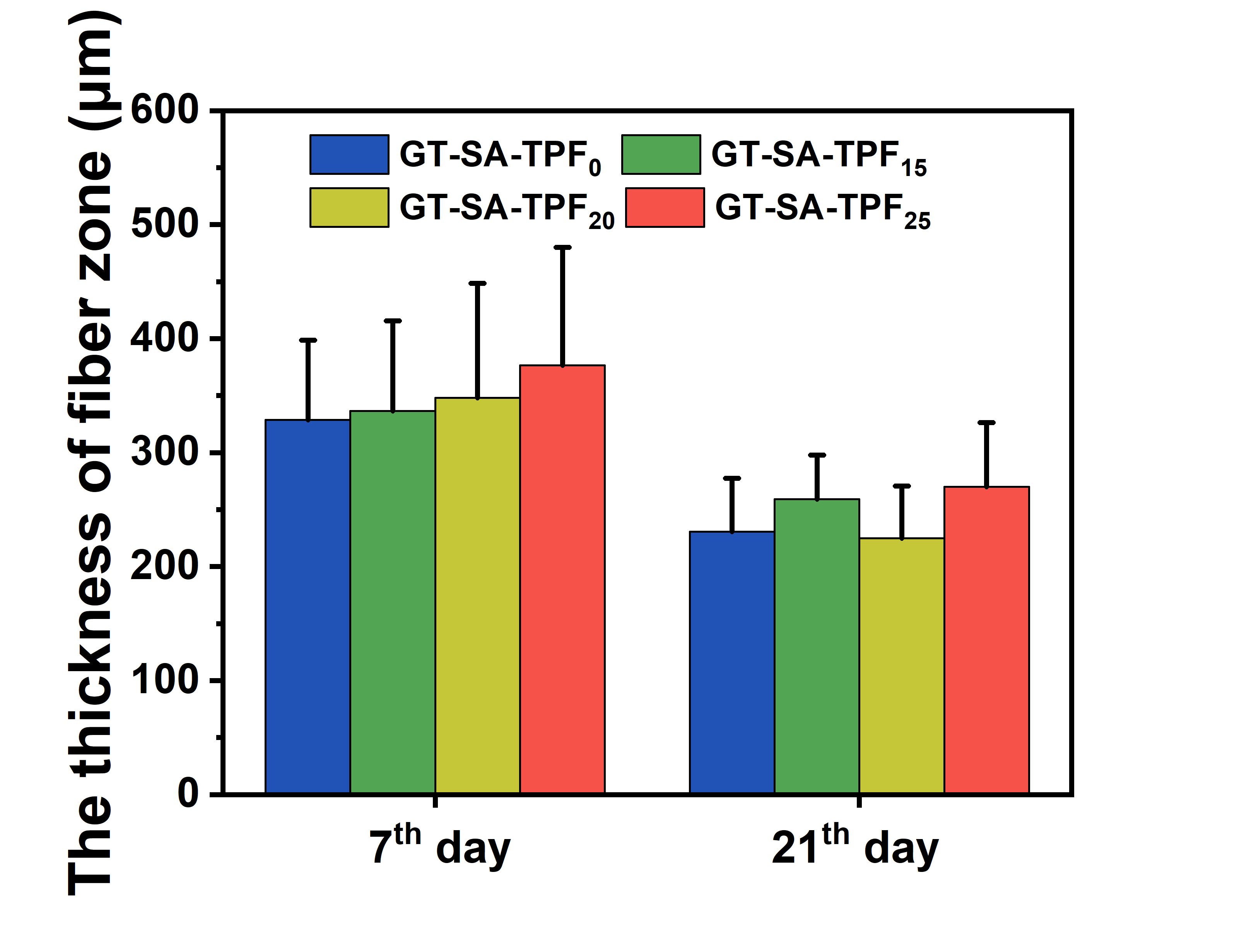
**

**Figure S7.** The thickness of the fiber zone of the skin tissues after implanted with hydrogels.


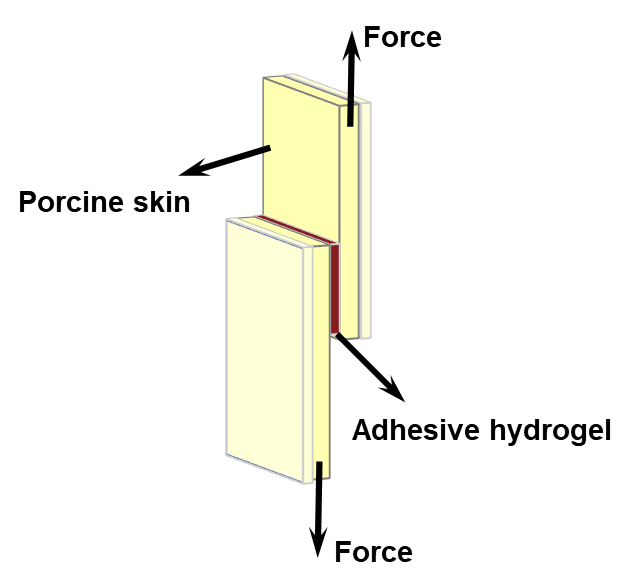


**Figure S8.** Illustration for the lap shear test.


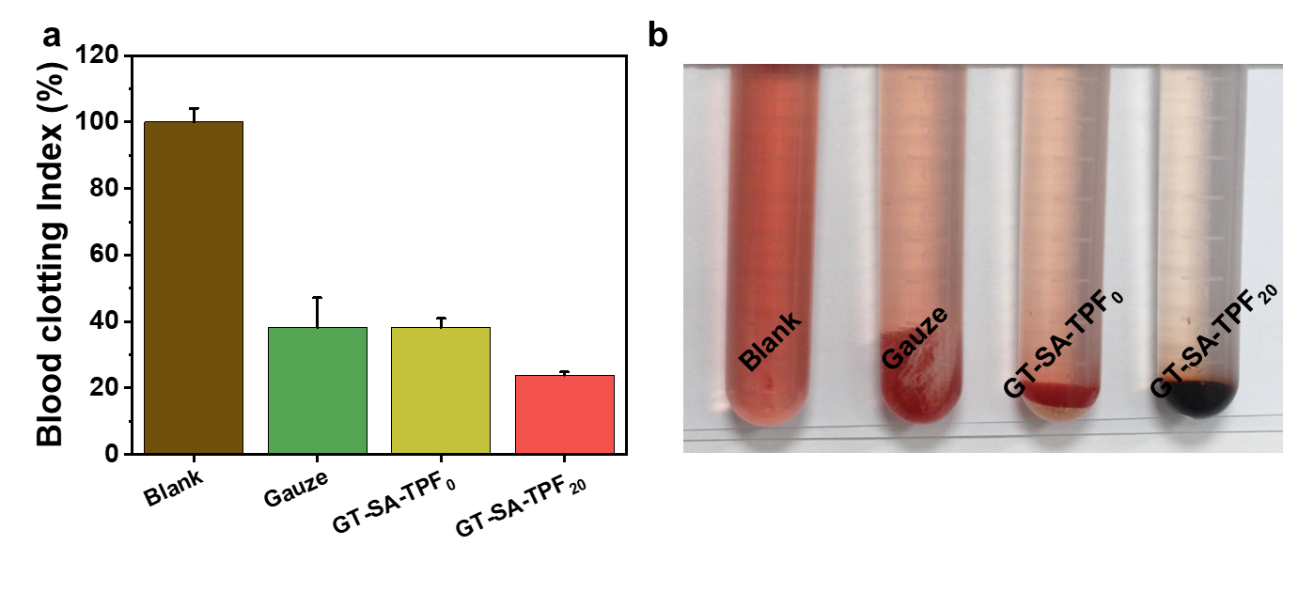


**Figure S9.** The blood clotting capacity evaluation. **a** Blood clotting index and **b** the images of the samples.


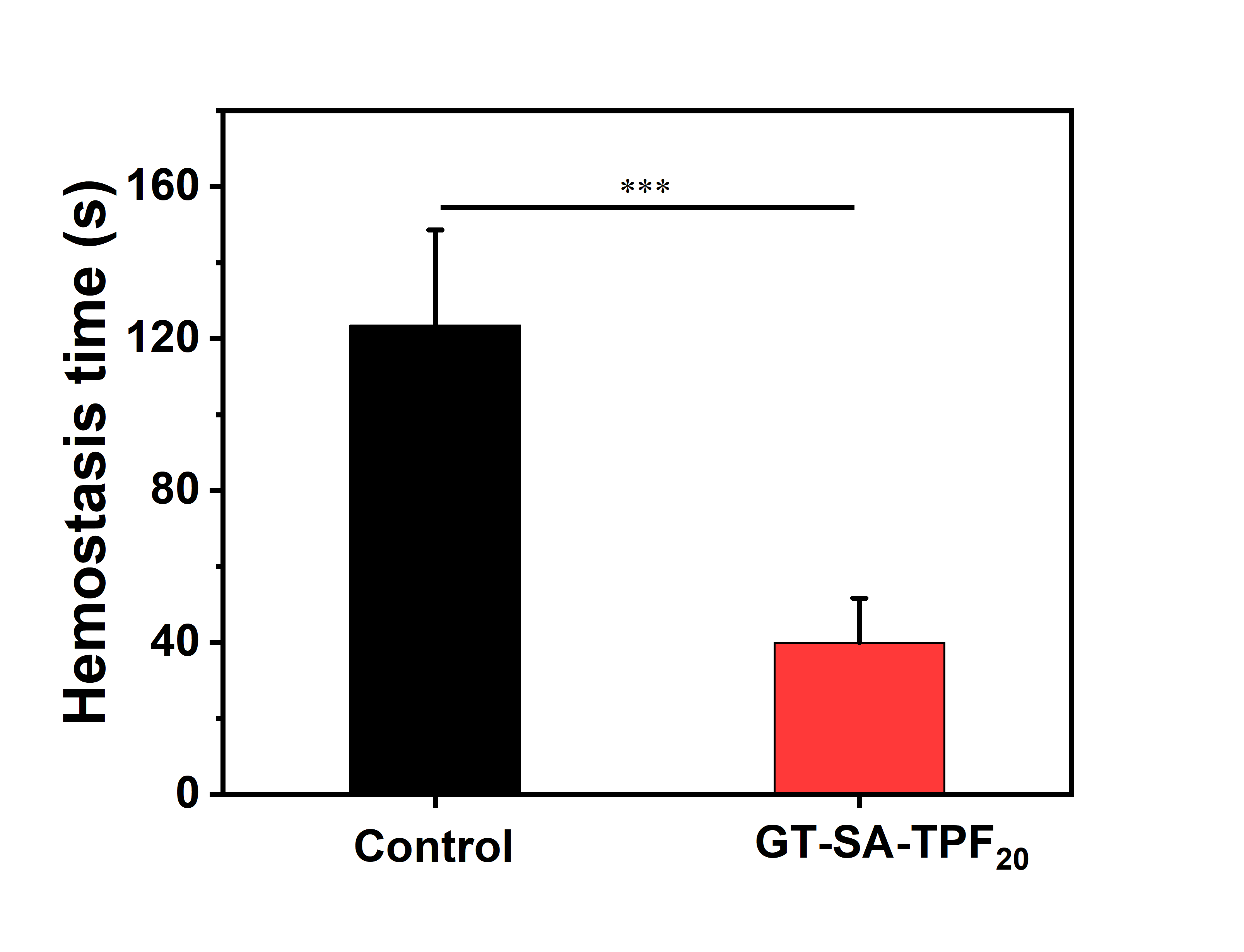


**Figure S10.** The hemostasis time in the liver in the mouse liver hemostasis test.


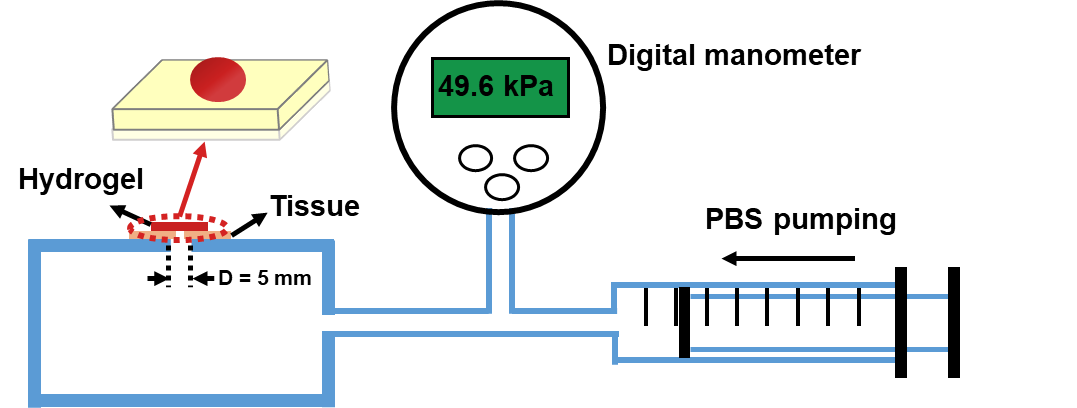


**Figure S11.** The illustration of the experimental equipment for the detection of burst pressure of the samples.

**Reference**

1. Liang, Y.; Li, Z.; Huang, Y.; Yu, R.; Guo, B., Dual-Dynamic-Bond Cross-Linked Antibacterial Adhesive Hydrogel Sealants with On-Demand Removability for Post-Wound-Closure and Infected Wound Healing. ACS Nano **15** (4), 7078-7093 (2021). <https://doi.org/10.1021/acsnano.1c00204>

2. Liang, Y.; Zhao, X.; Hu, T.; Chen, B.; Yin, Z., et al., Adhesive Hemostatic Conducting Injectable Composite Hydrogels with Sustained Drug Release and Photothermal Antibacterial Activity to Promote Full-Thickness Skin Regeneration During Wound Healing. Small **15** (12), 1900046 (2019). <https://doi.org/10.1002/smll.201900046>

3. Zhao, X.; Liang, Y.; Huang, Y.; He, J.; Han, Y., et al., Physical Double-Network Hydrogel Adhesives with Rapid Shape Adaptability, Fast Self-Healing, Antioxidant and NIR/pH Stimulus-Responsiveness for Multidrug-Resistant Bacterial Infection and Removable Wound Dressing. Adv. Funct. Mater. **30** (17), 1910748 (2020). <https://doi.org/10.1002/adfm.201910748>

4. Li, S.; Chen, N.; Li, X.; Li, Y.; Xie, Z., et al., Bioinspired Double-Dynamic-Bond Crosslinked Bioadhesive Enables Post-Wound Closure Care. Adv. Funct. Mater. **30** (17), 2000130 (2020). <https://doi.org/10.1002/adfm.202000130>

5. Zhou, L.; Dai, C.; Fan, L.; Jiang, Y.; Liu, C., et al., Injectable Self-Healing Natural Biopolymer-Based Hydrogel Adhesive with Thermoresponsive Reversible Adhesion for Minimally Invasive Surgery. Adv. Funct. Mater. **31** (14), 2007457 (2021). <https://doi.org/10.1002/adfm.202007457>

6. Peng, X.; Xu, X.; Deng, Y.; Xie, X.; Xu, L., et al., Ultrafast Self-Gelling and Wet Adhesive Powder for Acute Hemostasis and Wound Healing. Adv. Funct. Mater. **31** (33), 2102583 (2021). <https://doi.org/10.1002/adfm.202102583>
